# ﻿Expanding the knowledge of the bat fauna of the Brazilian Caatinga: new geographical records of molossid bats (Chiroptera, Molossidae) for the Chapada Diamantina region, with taxonomic notes

**DOI:** 10.3897/zookeys.1210.128570

**Published:** 2024-08-29

**Authors:** Franger J. García, José Ochoa-G, José L. Poma-Urey, Bruce W. Miller, Fábio C. Falcão, Martín Roberto del Valle Alvarez

**Affiliations:** 1 Programa de Pós-graduação em Zoologia, Universidade Estadual de Santa Cruz, Ilhéus, Brazil; 2 Centro de Estudios en Zoología Aplicada (CEZA), Facultad de Ciencias y Tecnología, Universidad de Carabobo, Valencia, Venezuela; 3 Área de Zoología, Museo de Historia Natural Noel Kempff Mercado de la Universidad Autónoma Gabriel René Moreno, Santa Cruz, Bolivia; 4 Neotropical Bat Acoustic and Risk Assessments, 11384 Alpine Rd., Canadian Lakes, MI 49346, USA; 5 Tetrapoda Consultoria Ambiental Ltda, Ilhéus, Bahia, Brazil; 6 Coleção de Mamíferos “Alexandre Rodrigues Ferreira” (CMARF), Departamento de Ciências Biológicas, Universidade Estadual de Santa Cruz, Ilhéus, Brazil

**Keywords:** Distributional records, dry forests, insectivorous bats, morphological diversity, Neotropical Molossidae, northeastern Brazil

## Abstract

The Caatinga, an exclusive biome in Brazil, is the largest tropical dry forest area in the Americas. It is characterized by a semi-arid climate and various soils that harbor a great diversity of flora and fauna. Novel records of aerial insectivorous bat species in the family Molossidae in the Chapada Diamantina, northeastern Brazil are presented. The study is based on field sampling of 115 molossid bat specimens from six genera and 12 taxonomically confirmed species, along with four taxa requiring further evaluation for definitive species identification. All specimens were obtained using mist nets around a small freshwater lagoon surrounded by semideciduous dry forest. The verified genera were *Cynomops*, *Eumops*, *Molossops*, *Molossus*, *Neoplatymops*, and *Nyctinomops*. Our findings enhance the understanding of bat diversity in the Brazilian Caatinga, with the first records of *Eumopsdelticus*, *E.bonariensis*, and *Molossuscurrentium*. The most abundant species were *Molossusrufus*, *Eumopsglaucinus*, *Cynomopsplanirostris*, *Nyctinomopslaticaudatus*, and *Molossusmolossus*. Previously unreported morphological and morphometric variations for these Caatinga taxa were examined. Additionally, information on sexual dimorphism in craniodental characteristics of *Molossopstemminckii* and variations in the presence of the sagittal crest in *Neoplatymopsmattogrossensis* are provided. Based on the voucher specimens from this study, the recognized number of species of Molossidae known from the Caatinga has increased to 21. Our results offer new insights into the taxonomy and biogeography of Neotropical molossids, highlighting their importance as members of bat communities in dry forest ecosystems from northeastern South America.

## ﻿Introduction

The Caatinga is an exclusive biome in Brazil and is considered the largest area of tropical dry forests in the Americas (Gutiérrez and Marinho-Filho 2017). This biome is characterized by a semi-arid climate with a wide variety of soils that harbor a great diversity of flora and fauna, including many endemic taxa (Garcia et al. 2014; Gutiérrez and Marinho-Filho 2017).

After Colombia, with 217 bat species (Rosero-Taramuel et al. 2023), Brazil has the second-highest richness of mammals belonging to the order Chiroptera (186) in the New World (Barros and Bernard 2023), with nearly 15% of the world’s bats (Bernard et al. 2011; Garbino et al. 2024). In the Caatinga, a range of 69 to 95 bat species have been reported (Garcia et al. 2014; Carvalho-Neto et al. 2017; Carmignotto and Astúa 2017; Silva et al. 2018), representing approximately 53% of the bat diversity of the country (Delgado-Jaramillo et al. 2020). Compared to other biomes comprised of the dry diagonal of non-forest formations in South America, the Caatinga stands out for its significant bat diversity, especially insectivores (Gregorin et al. 2008; Silva and Bernard 2017).

New World free-tailed bats (Molossidae) are a diverse family of aerial insectivorous species predominantly occurring in tropical and subtropical regions, with limited species diversity in temperate zones (Simmons 2005). They are considered the most highly adapted aerial foragers, specifically hawking high-flying insects (Norberg and Rayner 1987). Morphologically, they have relatively long and narrow wings with a high aspect ratio and wing loading. Their flight patterns include high-speed flight in open spaces with relatively low maneuverability (Norberg and Rayner 1987; Jung et al. 2014).

Previously, the diversity of molossids in the Caatinga was estimated to range between 14 and 17 species, based on recent checklists (Carvalho-Neto et al. 2017; Silva et al. 2018). However, many of those reported taxa require a careful, and detailed review of the captured individuals due to recent nomenclatural changes and new species descriptions (Gregorin et al. 2016; Loureiro et al. 2018a; Arenas-Viveros et al. 2021). Additionally, it is necessary to verify the taxonomic identification of many cryptic taxa (Bernardi et al. 2009; Loureiro et al. 2018b), given the lack of comprehensively verified voucher specimens represents a challenge in recognizing intra-specific variations.

As aerial insectivorous, most molossids are known to forage over forest canopies and open spaces, flying from medium to high strata where they feed on relatively large prey (Jung et al. 2014). Given their foraging preferences and feeding strategies, they are difficult to capture with conventional techniques, usually set at ground level (mist nets and harp traps). However, mist nets and harp traps can more effectively capture molossids at the canopy level. As a result, many of the Neotropical aerial insectivorous bats, including most of the molossids are poorly represented in field studies or as vouchers in zoological collections, and biological information is scarce (Ochoa-G et al. 2000; Moras et al. 2018; Miller et al. 2023).

As part of an evaluation of the bat community occurring within the Caatinga, an important number of molossids were captured during nine nights of mist net sampling at a single location associated with a freshwater lagoon. The results of this study confirm the taxonomic richness of this family in the region and provide additional data on an important number of species considered “cryptic” (Bernardi et al. 2009; Gregorin 2009) and highly variable in their morphological and physiological characteristics (Loureiro et al. 2018b); some of these taxa have not been reported in previous publications related to the Caatinga biome (Silva et al. 2018). We underscore the importance of accurately documenting voucher specimens in scientific collections to corroborate taxa identification for future works (e.g., Poma-Urey et al. 2023).

## ﻿Materials and methods

**Figure 1.**
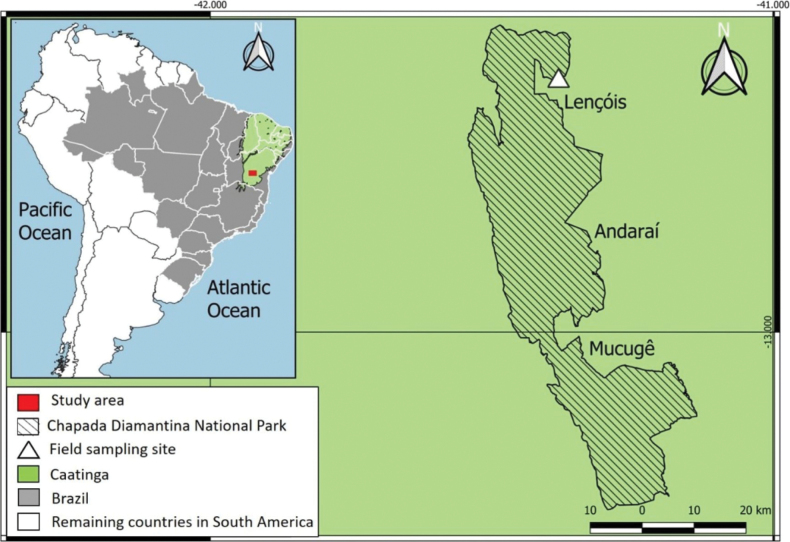
Map of Chapada Diamantina National Park, northeastern Brazil, showing the geographic location of the sampling site in Lençóis.

The survey was conducted within the Caatinga biome in Lençóis, Chapada Diamantina region, Bahia State, northeastern Brazil (12.54822°S, 41.37928°W, 351 m. a.s.l., Fig. 1). The dominant vegetation of the study area corresponds to a primary semideciduous forest. All geographic coordinates are in decimal degrees with the base datum WGS84.

**Figure 2.**
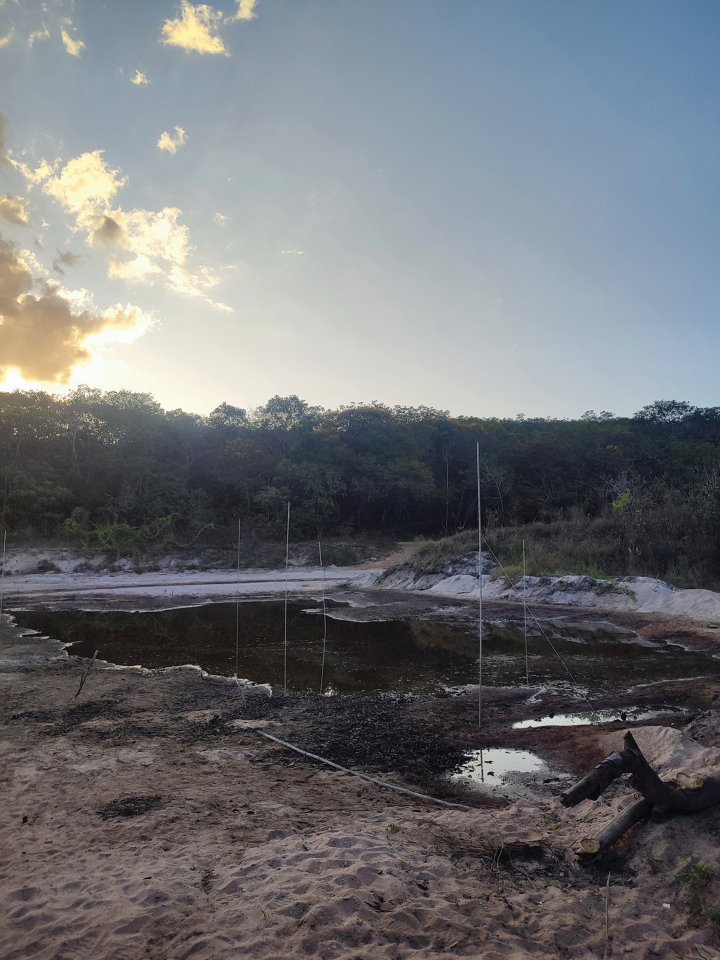
Freshwater lagoon used as capture site of the molossid bats reported in this study for Lençóis (Chapada Diamantina National Park). Associated vegetation corresponds to a primary semideciduous forest.

Fieldwork was conducted from 6 to 14 December 2023 using seven mist nets of varying sizes (6 × 3 m, 9 × 3 m, and 12 × 3 m) set around a freshwater lagoon formed by the drying of an intermittent river within the Chapada Diamantina National Park (Fig. 2).

All bats were captured following the guidelines of Sikes (2016), and those retained as vouchers were humanly euthanized (injecting a barbiturate anesthetic via intraperitoneal), then fixed in formaldehyde (10%), and preserved in ethanol (70%). All handling protocols were approved by the Animal Use Ethics Committee of the Universidade Estadual de Santa Cruz (CEUA-UESC Nos. 004/2020 and 024/2021). All capture and collecting were under permit number 17131-4, provided by the Sistema de Autorização e Informação em Biodiversidade – SISBIO of the Instituto Chico Mendes de Conservação da Biodiversidade – ICMBio, Brazil; this permit was granted to one of the researchers (MRAV). The following authorization allowed us to conduct fieldwork within the study area: ICMBio – SISBIO 79060-1 for PARNA Chapada Diamantina. Vouchers were deposited in the Coleção de Mamíferos Alexandre Rodrigues Ferreira (**CMARF**) at the Universidade Estadual de Santa Cruz (**UESC**), Bahia, Brazil. All skulls were extracted and measured to compare cranial morphology and verify identifications.

Species-level identifications were made by the authors, who have extensive experience with the taxonomy of Neotropical bats and the management of natural history collections. We did not include a review of voucher specimens deposited in local collections for this study. This was primarily because most collections related to specimens from the Caatinga have incomplete representation of species in the family Molossidae. However, we did a comprehensive review of the literature to verify the accuracy of our identifications. Secondly, as these included cryptic species, many identifications of local collection-based specimens could be in error as most Latin American institutions do not have specialists for this group. Therefore, all the identifications reported here are based on our broad taxonomic knowledge and supported by published sources.

Preliminary identifications were made based on keys for Neotropical bats (Eger 2008; Díaz et al. 2016, 2021), as well as those specifically for species of Molossidae occurring in Brazil (Gregorin and Taddei 2002; Loureiro et al. 2018b). To verify the preliminary identifications, qualitative and quantitative characteristics were compared to comprehensive resources related to the taxa of molossid bats. These included taxonomic revisions, descriptions of new species, and phylogenetic reviews of the genera *Eumops* (Marinkelle 1970; Eger 1977; Freeman 1981; Bernardi et al. 2009; Gregorin 2009; Gregorin and Cirranello 2015; Gregorin et al. 2016; Ruelas and Soria 2021), *Molossus* (Freeman 1981; Dolan 1989; Gregorin and Cirranello 2015; Loureiro et al. 2018a, 2018b), *Cynomops* (Gregorin and Cirranello 2015; Moras et al. 2018), *Molossops* (Gamboa Alurralde and Díaz 2019; Ramírez-Chaves et al. 2023), *Nyctinomops* (Bianconi et al. 2009; Gregorin and Cirranello 2015; Rocha et al. 2015; Oliveira et al. 2019; Portugal-Zegarra et al. 2020; Barquez et al. 2023), and *Neoplatymops* (Sazima and Taddei 1976; Willig 1985; Willig and Jones 1985; Gregorin and Cirranello 2015).

The following morphological parameters previously reported for molossids (Bernardi et al. 2009; Gregorin et al. 2016; Loureiro et al. 2018b; Moras et al. 2018; Ruelas and Soria 2021), were considered: total length of body (**TLB**); tail length (**TL**); length of hind limb (**LHL**); ear length (**EL**); weight (**W**); forearm length (**FA**); greater length of skull with incisors (**GSLI**); greater length of skull excluding incisors (**GSL**); condylobasal length (**CBL**); condylo-incisor length (**CIL**); palatal length (**PL**); zygomatic breadth (**ZB**); mastoidal breadth (**MB**); braincase width (**BCW**); interorbital width (**IOW**); length of maxillary toothrow (**C-M3**); width across upper canines (**C-C**); width across upper molars (**M3-M3**); greatest length of mandible (**LM**); and length of mandibular toothrow (**c-m3**). All measurements are in millimeters and mass in grams.

Due to the pronounced sexual dimorphism reported for members of the family Molossidae (Eger 2008; Gregorin et al. 2011; Moras et al. 2018; Loureiro et al. 2018b), principally body and cranial dimensions, morphometric analysis was conducted separately for males and females. The results are presented for each species based on a structure that includes sex, CMARF catalog number, external and cranial measurements by sex, morphological variations, and a brief description of the diagnostic traits used to verify identification. All bats included in the diagnosis were adults unless otherwise noted.

## ﻿Results

Of the 335 bats captured during this survey, 115 were molossids, of which 102 were identified to the species level, and 13 were equivocal and only identified to genus. Six genera were recorded (*Cynomops* Thomas, 1920; *Eumops* Miller, 1906; *Molossops* Peters, 1866; *Molossus* É. Geoffroy St.-Hilaire, 1805; *Neoplatymops* Peterson, 1965, and *Nyctinomops* Miller, 1902) and 12 confirmed species.

Sampled specimens included notable representative series of *Molossusrufus* (26 spcms), *Eumopsglaucinus* (21 spcms), *Molossusmolossus* (15 spcms), *Cynomopsplanirostris* (10 spcms), *Nyctinomopslaticaudatus* (eight spcms), *Neoplatymopsmattogrossensis* (seven spcms), and *Molossusaztecus* (five spcms). The remaining captures corresponded to five specimens of *Molossuscurrentium*, two medium-sized specimens with morphological and metric characteristics documented for the *Eumopsbonariensis* complex, two *Molossopstemminckii* specimens, and one *Nyctinomopsmacrotis* specimen. Several captured individuals did not match previously known species and only were identified as morphospecies. They include one specimen consistent with the genus *Molossops* (referred to here as *Molossops* sp.) and three morphotypes whose characteristics correspond to the genus *Molossus* (*Molossus* sp. 1, 2, and 3).

### ﻿Species accounts

#### ﻿ Cynomops planirostris

Taxon classificationAnimaliaChiropteraMolossidae

(W. Peters, 1866)

B36E9B43-E631-5324-83B8-6C3B085B1B9C

##### Summary of captures.

Six females (CMARF 2111–2116) and four males (CMARF 2117–2120).

##### External measurements and weights.

The average and range of external measurements and weights for females: TLB: 90.00 (80.00–97.00), TL: 31.00 (27.00–36.00), LHL: 5.97 (5.40–7.00), EL: 13.17 (13.00–14.00), W: 9.50 (9.00–10.00). Males: TLB: 90.00 (89.00–92.00), TL: 30.00 (28.00–32.00), LHL: 5.88 (5.34–6.01), EL: 13.80 (13.08–14.05), W: 9.75 (9.00–10.00).

##### Morphological description.

**Figure 3.**
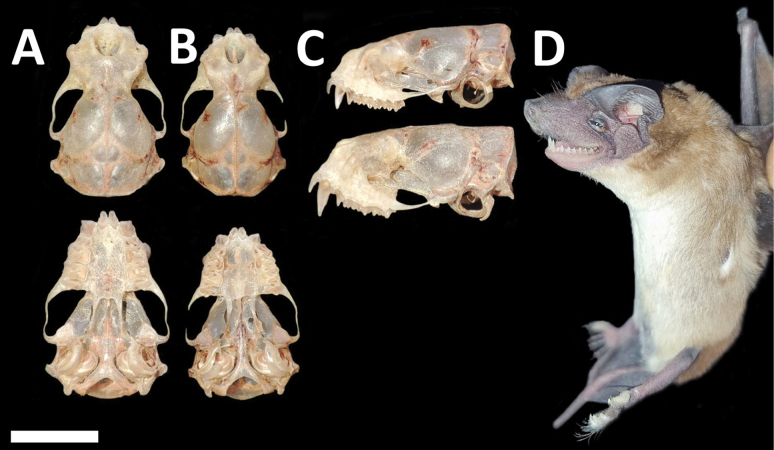
Dorsal, ventral, and lateral views of the skull of a male (CMARF 2118 **A, C** lower) and a female (CMARF 2115 **B, C** upper) of *Cynomopsplanirostris* collected in the Caatinga (northeastern Brazil), showing sexual dimorphism in some dimensions **D** external body features of a female of *Cynomopsplanirostris* from the same locality (CMARF 2115). Scale bar 5 mm.

Dorsal fur varies from chocolate brown to yellowish brown, contrasting with the paler ventral coloration. Skulls with a relatively low and short rostrum (Fig. 3). Lacrimal ridge conspicuous with the anterior face sloped smoothly to the forehead. Incisive foramina positioned closer to the accessory foramen and the three foramina (incisive and accessory) forming an equilateral triangle (seen with magnification from above). Basisphenoid pits are shallow. Values of some skull measurements for the females and males are shown in Table 1.

**Table 1.** External and cranial measurements for eight species of bats of the family Molossidae with confirmed identities and one unidentified morphospecies. Data based on specimens collected in the Caatinga biome (Lençóis, Chapada Diamantina region, northeastern Brazil), according to this study (catalog numbers are indicated) and previous information in the literature. Abbreviations are described in the Materials and methods.

TAXA	FA	GSLI	GSL	CBL	CIL	PL	ZB	MB	BCW	IOW	C-M3	C-C	M3-M3	LM	c-m3
*Cynomopsplanirostris*															
Female (CMARF 2111)	31.19	15.49	15.00	14.34	14.95	6.86	9.98	9.41	7.86	4.30	5.82	3.93	7.20	10.99	5.66
Female (CMARF 2112)	31.93	16.35	16.04	15.23	15.87	6.98	10.64	10.33	7.79	3.96	6.04	4.33	7.55	11.57	6.97
Female (CMARF 2113)	31.46	15.92	15.30	15.69	15.29	6.05	10.28	9.93	7.73	4.03	5.69	4.15	7.39	10.91	6.60
Female (CMARF 2114)	31.61	15.33	14.89	14.34	14.81	6.38	10.31	9.93	7.88	4.08	5.62	4.11	7.40	10.59	6.63
Female (CMARF 2115)	32.09	15.89	15.71	15.06	15.66	6.21	10.43	9.90	7.38	4.06	5.80	4.44	7.46	11.39	6.48
Female (CMARF 2116)	32.21	15.94	15.60	14.73	15.30	6.42	10.11	10.05	7.78	4.07	5.86	4.21	7.51	11.13	6.85
Male (CMARF 2117)	32.94	17.07	16.98	15.92	16.57	7.63	11.13	10.86	8.05	4.07	6.19	4.57	7.83	12.02	6.50
Male (CMARF 2118)	32.63	16.85	15.95	14.51	15.24	6.72	10.58	10.26	7.76	3.92	5.74	4.81	7.20	11.78	6.05
Male (CMARF 2119)	33.68	16.85	16.10	15.83	16.66	7.51	11.03	10.74	7.87	4.10	6.22	4.80	7.70	11.69	6.32
Male (CMARF 2120)	32.78	17.20	15.00	15.83	16.64	7.29	11.19	10.84	8.10	4.40	6.36	4.68	7.94	12.04	7.32
*Eumopsbonariensis*															
Female (CMARF 2121)	44.63	18.15	17.55	17.54	17.18	7.17	10.63	9.91	8.89	3.99	6.76	4.36	7.59	12.15	7.81
Female (Bernardi et al. 2009)	47.60	19.20	–	17.80	18.40	–	11.10	10.50	9.10	4.20	7.30	4.60	8.10	13.00	7.70
Female (Bernardi et al. 2009)	45.90	19.40	–	17.60	18.00	–	11.40	10.60	9.20	4.00	7.20	4.60	8.10	12.80	7.70
Female (Ruelas and Soria 2021)	49.30	18.86	–	18.09	18.72	7.80	11.69	10.45	9.01	4.29	7.50	0.96*	1.90*	14.48	8.78
*Eumopsdelticus*															
Female (CMARF 2122)	47.53	18.34	18.10	17.27	17.46	7.23	10.58	10.12	8.83	3.80	7.15	4.53	7.64	12.36	7.60
Female (Carter and Dolan 1978, Holotype: BMNH 23.8.9.7)	–	–	18.80	17.90	–	–	10.90	10.30	9.10	4.20	7.20	4.70	7.90	12.70	7.90
Female (Ruelas and Soria 2021)	47.68	18.38	–	–	18.11	7.13	11.08	10.17	8.78	4.42	6.93	–	–	13.18	7.79
*Eumopsglaucinus*															
Female (CMARF 2123)	60.98	24.08	23.60	22.21	23.22	9.08	14.41	13.05	11.62	5.02	9.26	5.65	10.26	17.29	10.47
Female (CMARF 2124)	56.54	23.70	22.80	21.41	22.74	10.35	13.90	12.89	10.69	4.82	8.93	5.80	9.92	16.46	10.26
Female (CMARF 2125)	60.20	23.82	23.50	22.26	23.52	10.09	14.52	12.96	11.12	4.94	8.91	5.83	10.05	17.50	10.66
Female (CMARF 2126)	59.22	24.45	23.99	22.38	23.41	10.66	14.30	12.92	11.30	5.02	9.27	5.92	10.02	17.32	10.63
Female (CMARF 2127)	60.80	24.43	23.78	22.53	23.61	10.29	14.39	13.11	11.28	5.04	9.30	5.81	9.97	17.53	10.49
Female (CMARF 2128)	60.45	23.90	23.50	22.07	23.32	10.16	14.13	12.60	11.02	5.17	9.22	5.65	9.97	17.35	10.52
Female (CMARF 2129)	62.61	23.93	23.66	22.09	23.16	10.45	14.87	13.11	11.57	4.93	8.81	5.91	10.33	17.14	10.23
Female (CMARF 2130)	63.23	24.12	23.78	22.17	23.27	10.03	14.27	12.95	11.37	4.96	9.01	5.65	10.05	17.15	10.72
Male (CMARF 2131)	59.41	24.43	24.17	22.49	23.99	10.45	14.86	13.74	11.25	5.17	9.55	6.38	9.55	18.15	10.97
Male (CMARF 2132)	59.13	24.45	24.25	22.79	24.03	10.26	14.45	13.14	11.29	4.88	9.15	6.12	9.78	17.35	10.65
Male (CMARF 2133)	58.50	24.78	23.99	22.57	23.94	10.66	14.37	12.86	11.19	4.98	8.82	5.72	9.53	17.21	10.43
Male (CMARF 2134)	60.02	24.63	24.10	22.60	24.01	10.21	14.82	13.56	11.13	5.31	9.37	5.82	10.33	17.72	10.70
Male (CMARF 2135)	60.20	24.66	23.95	22.85	23.80	9.94	15.34	13.51	11.59	5.13	9.21	6.14	10.01	17.28	10.50
Male (CMARF 2136)	59.74	24.30	23.93	22.65	23.78	10.15	15.20	13.30	11.24	4.90	9.54	5.95	10.15	17.53	10.24
Male (CMARF 2137)	58.55	24.38	23.80	24.42	23.74	10.00	14.54	13.25	10.86	4.87	8.86	6.04	9.79	17.43	10.20
Male (CMARF 2138)	59.65	24.68	24.10	22.61	24.00	10.35	14.48	13.34	11.37	5.26	9.29	6.01	10.40	17.46	10.75
Male (CMARF 2139)	64.33	24.50	24.20	23.23	24.17	10.38	14.39	13.21	11.31	4.96	9.12	6.47	10.30	17.50	10.90
Male (CMARF 2140)	64.16	23.81	23.66	22.30	23.26	10.08	14.16	12.87	10.71	4.98	8.84	5.78	10.17	17.11	10.11
Male (CMARF 2141)	59.75	24.69	24.50	22.76	24.25	10.99	14.34	13.11	10.92	4.96	9.57	5.59	10.12	17.81	10.92
Male (CMARF 2142)	52.38	24.63	23.86	22.67	23.76	10.57	14.62	13.28	11.45	5.43	9.39	5.85	9.92	17.15	10.67
Male (CMARF 2143)	59.28	24.55	24.00	22.59	23.96	10.70	14.95	13.35	11.25	5.18	9.18	5.87	10.11	17.51	10.35
*Molossopstemminckii*															
Female (CMARF 2144)	31.53	14.27	14.00	13.38	13.83	6.05	9.21	8.73	6.86	3.73	5.35	3.75	6.64	10.03	5.84
Male (CMARF 2145)	32.33	14.85	13.50	13.94	14.52	6.66	9.44	8.72	7.25	3.78	5.51	4.32	6.71	10.86	6.19
*Molossops* sp.															
Female (CMARF 2146)	31.73	14.29	14.00	13.44	13.76	6.28	9.51	8.58	7.22	3.78	5.07	4.17	6.69	10.15	5.91
*Neoplatymopsmattogrossensis*															
Female (CMARF 2210)	29.39	14.64	14.40	13.62	14.30	6.70	–	9.27	7.06	3.69	5.40	3.70	6.59	9.92	5.80
Female (CMARF 2211)	29.39	14.34	14.21	13.60	14.19	6.70	–	8.51	6.78	3.42	5.49	3.47	6.52	9.52	5.97
Female (CMARF 2212)	30.01	14.85	14.40	13.71	14.30	6.61	9.56	8.98	7.14	3.55	5.56	3.52	6.87	9.84	5.84
Female (CMARF 2213)	29.71	14.62	14.18	13.42	14.12	6.82	9.29	8.52	6.88	3.51	5.14	3.70	6.68	10.08	5.72
Female (CMARF 2214)	24.31	14.62	14.28	13.52	14.08	6.49	9.91	8.65	7.47	3.59	5.21	3.74	6.69	9.48	5.91
Female (CMARF 2215)	23.69	14.47	14.20	13.51	14.13	6.31	9.21	8.63	7.26	3.54	5.21	3.77	6.76	9.92	5.75
Female (CMARF 2216)	28.69	14.01	13.99	13.32	13.96	6.46	9.47	8.96	7.06	3.68	5.18	3.73	6.54	9.78	5.67
*Nyctinomopslaticaudatus*															
Female (CMARF 2217)	44.26	17.93	17.00	15.95	16.78	7.35	10.07	9.57	8.51	3.50	6.55	3.89	7.68	11.64	7.11
Female (CMARF 2218)	45.90	18.10	17.00	15.78	16.68	6.54	10.25	9.47	8.69	3.50	6.17	3.93	7.48	12.17	7.56
Female (CMARF 2219)	44.02	17.70	17.05	15.87	16.58	6.95	10.21	9.67	8.46	3.75	6.54	3.91	7.33	11.83	7.10
Female (CMARF 2220)	44.97	17.43	17.10	15.44	16.19	6.14	9.66	9.65	8.20	3.65	6.09	3.68	7.07	11.91	7.16
Female (CMARF 2221)	44.47	18.16	17.00	16.00	16.95	7.02	10.39	9.58	8.72	3.64	6.67	3.93	7.63	12.15	7.43
Female (CMARF 2222)	44.67	17.61	17.00	15.56	16.34	6.95	10.19	9.86	8.48	3.57	6.44	3.75	7.42	11.68	6.72
Male (CMARF 2223)	44.47	17.78	16.98	16.03	16.57	7.26	9.74	9.37	8.38	3.37	6.27	3.66	7.16	12.01	7.23
Male (CMARF 2224)	45.19	18.62	17.96	16.41	17.23	7.70	10.43	10.03	8.65	3.54	6.67	4.00	7.39	12.17	7.30
*Nyctinomopsmacrotis*															
Female (CMARF 2225)	59.83	23.36	22.68	21.38	22.29	9.46	12.54	11.80	10.34	4.35	8.27	4.91	8.63	16.27	9.31

*Variable incorrectly measured.

##### Identification.

Externally, the forearm length (< 40 mm) and the ventral coloration paler than the dorsum in all specimens, in addition to cranial measurements and the arrangement of incisive and accessory foramina in the shape of an equilateral triangle, distinguish this species from its most similar congeners in the area (*Cynomopsgreenhalli* Goodwin, 1958, and *C.milleri* (Osgood, 1914), Moras et al. 2018; López Berrizbeitia and Díaz 2021).

#### ﻿ Eumops bonariensis

Taxon classificationAnimaliaChiropteraMolossidae

(W. Peters, 1874)

01534CCE-20B9-5EEC-B6A0-3EEDB11EB90F

##### Summary of captures.

One female (CMARF 2121).

##### External measurements and weight.

TLB: 108.00, TL: 43.00, LHL: 7.00, EL: 18.00, W: 10.00.

##### Morphological description.

**Figure 4.**
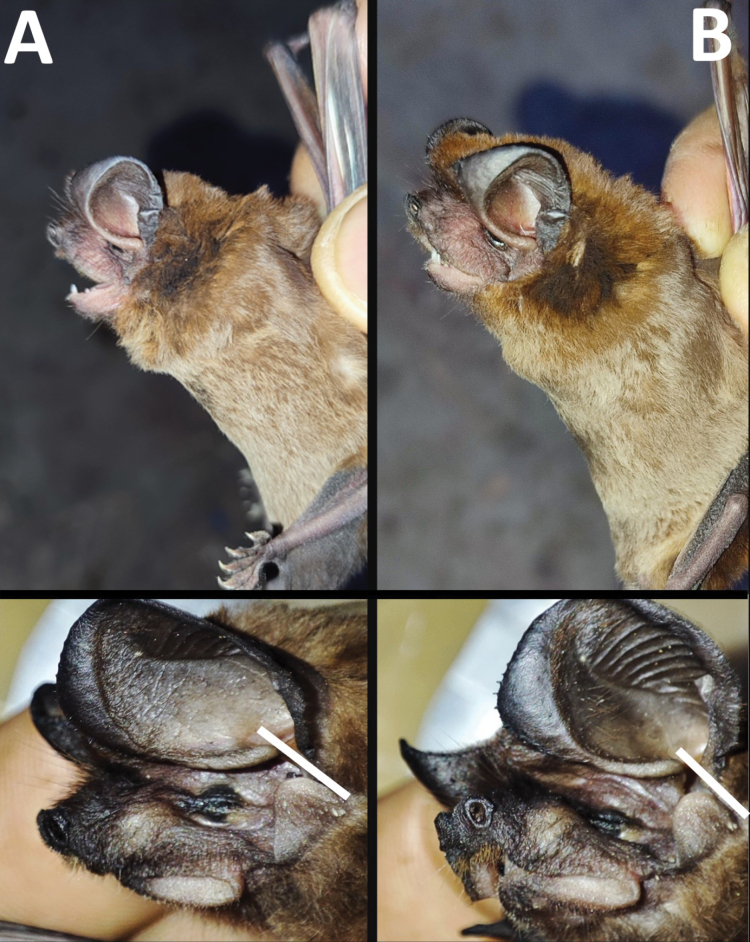
Females of *Eumopsbonariensis* (CMARF 2121; **A**) and *Eumopsdelticus* (CMARF 2122; **B**) showing the body coloration, the antitragus shape, and the extension of the internal ear keel that reaches the posterior part of the antitragus (indicated by the white bar).

Dorsal fur is chocolate brown, with the basal portion of hairs paler than the tips. Ventral and dorsal coloration show slight contrast, with the hairs around the neck and shoulders darker than the rest (Fig. 4). Upper lips are slightly wrinkled. The upper border of the narial region is surrounded by small and obtuse warts. Small hairs cover internarial ribs. Ears broad, rounded, and joined in a common point. The upper border of ears with pointed and fleshy warts. The inner keel of the ears reaching the antitragus, not extending beyond the posterior part of this structure. Antitragus long and semicircular. The tragus is slightly squared, with the upper extreme narrower, giving the appearance of an obelisk shape.

**Figure 5.**
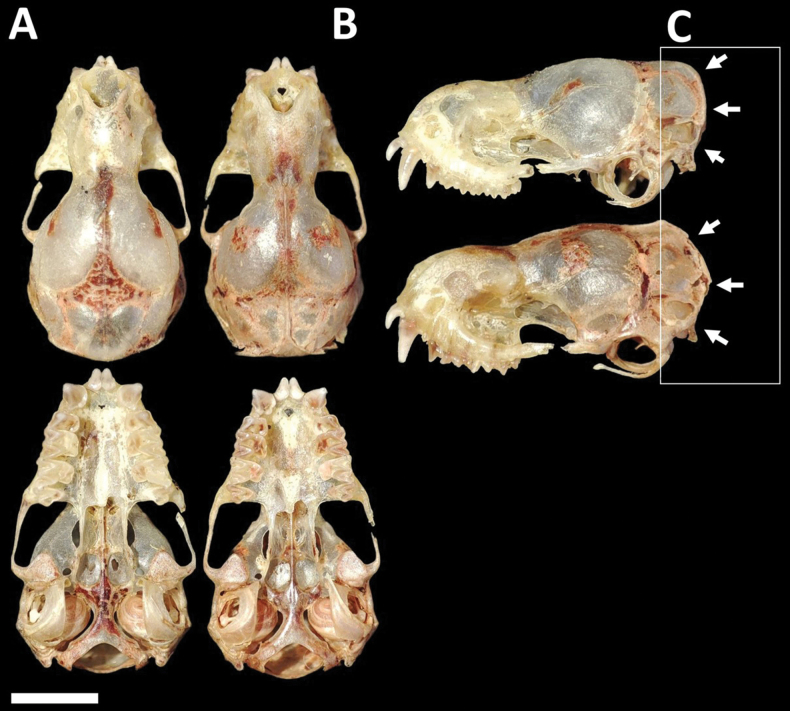
Dorsal, ventral, and lateral views of the skulls of two species of *Eumops* collected in the Caatinga, northeastern Brazil. **A, C** (upper)- *Eumopsbonariensis* (CMARF 2121) **B, C** (lower)- *Eumopsdelticus* (CMARF 2122). White arrows indicate the morphological differences between both taxa, highlighted in the species’ account. Scale bar 5 mm.

**Figure 6.**
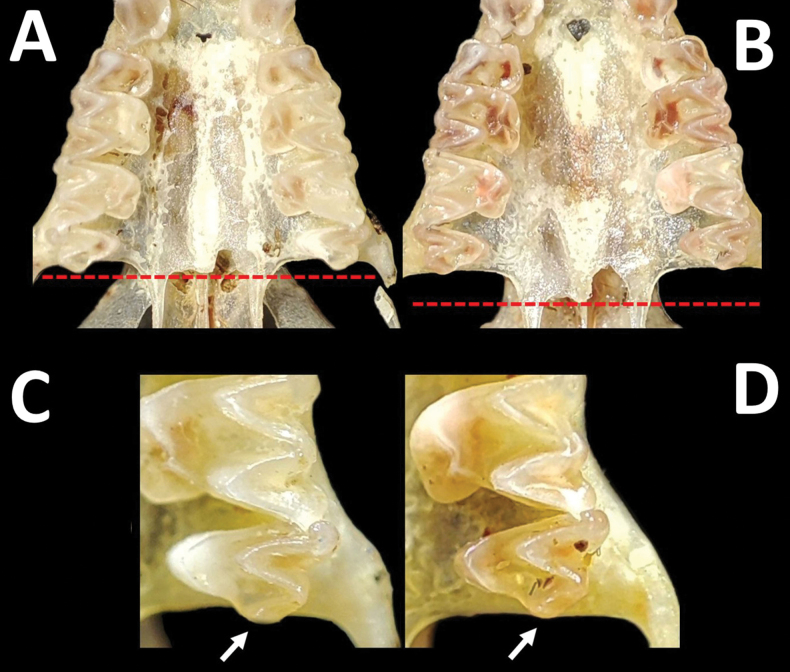
Enlarged views (not to scale) of some cranial and dental characteristics of *Eumopsbonariensis* (**A, C**) and *Eumopsdelticus* (**B, D**). The discontinuous red lines and white arrows indicate the differences in palatal and maxillary lengths between both species, respectively.

The skull is broader across the rostrum, with an evident depression near the mastoid bone; the braincase is deeper (globular shape), and the lateral region is curved (Fig. 5). Interparietal bones are not elongated and not visible in the lateral view of the skull. The sagittal crest is less developed than the lambdoidal crest. Upper incisors with divergent tips projected forward, forming an angle with the canines of 45°. Upper canines and first premolars in contact. The second upper premolar is protocone-wide and robust (Fig. 5). The third upper molar has a well-developed commissure. The posterior part extends beyond the maxillary bone (Fig. 6). The palate does not extend beyond the level of the third upper molars (Fig. 6). Mesopterygoid fossa with basisphenoid pits deep, narrow anteriorly and broader posteriorly, with an oval shape (Fig. 5). Rib between basisphenoid pit wide. Incisive foramen diminutive. The mandible is slender, with an articular process wider and more developed than the condylar and coronoid processes. Lower incisors bilobed. Values of some cranial measurements of the collected female and the data for the same variables provided by other studies are shown in Table 1.

##### Identification.

Externally, the forearm length (> 40 mm) and the shape and depth of the basisphenoid pits (deep, narrowing anteriorly, and broader posteriorly) separate this species from *Eumopshansae* Sanborn, 1932 (Eger 2008). Compared to the closest morphologically similar species, *Eumopspatagonicus* O. Thomas, 1924, and *Eumopsdelticus* O. Thomas, 1923, *E.bonariensis* can be differentiated from *E.patagonicus* by being larger in some external and cranial measurements, including the lengths of the forearm and skull. Additionally, the extension of the internal keel of the ears, not reaching the anterior antitragus area in *E.patagonicus*, is a discreet characteristic that can help differentiate *E.patagonicus* from *E.bonariensis* (Díaz et al. 2021). However, Bernardi et al. (2009) identified an individual as *E.patagonicus* with an internal keel reaching the middle region of the antitragus. With respect to *E.delticus*, cranial morphological characters are provided in the Discussion to aid in distinguishing both species.

#### ﻿ Eumops delticus

Taxon classificationAnimaliaChiropteraMolossidae

O. Thomas, 1923

57F9B6ED-8E54-5888-AF84-FF6D544E5B47

##### Summary of captures.

One female (CMARF 2122).

##### External measurements and weight.

TLB: 107.00, TL: 42.00, LHL: 6.00, EL: 19.00, W: 11.00.

##### Morphological description.

Dorsal fur is cinnamon brown, with the basal portion of the hairs paler than the tips. The color of ventral and dorsal fur shows slight contrast, with the hairs around the neck and shoulders darker than the rest (Fig. 4). The upper lips are slightly wrinkled. The upper border of the nares is surrounded by small and obtuse warts. Small hairs cover internarial ribs. Ears broad, rounded, and joined at a common point. The upper border of ears with pointed and fleshy warts. The inner keel of the ears reaching the antitragus, not extending beyond the posterior part of this structure. Antitragus long and semicircular. The tragus is slightly subquadrate, with the upper extreme narrower, giving an obelisk appearance.

The skull is broader across the rostrum with an evident depression near the mastoid bone, the braincase is deeper (globular shape), and the lateral region is curved (Fig. 5). Interparietal bones are elongated, clearly visible in the lateral view of the skull. Sagittal and lambdoidal crests developed. Upper incisors with divergent tips projected forward, forming an angle of 45° with the canines. Upper canines and first premolars in contact. The second upper premolars have protocones that are thin and not robust (Fig. 5). The third upper molars have a well-developed commissure. The posterior part of the third upper molar does not extend beyond the maxillary bone (Fig. 6). Palate extends beyond the level of the third upper molar (Fig. 6). Mesopterygoid fossa deep and narrower anteriorly, with basisphenoid pits deep, wider posteriorly and oval in shape (Fig. 5). Rib between basisphenoid pit thin. Incisive foramen large. The mandible is slender, with an articular process that is wider and more developed than the condylar and coronoid processes. Lower incisors are bilobed. The values of some cranial measurements of the female collected and comparable data from other studies, are shown in Table 1.

##### Identification.

Externally, the forearm length (> 40 mm) and the shape and depth of the basisphenoid pits (deep and wider posteriorly) separate this species from *Eumopshansae* (Eger 2008). When comparing differences to the closest congeners morphologically, *Eumopspatagonicus* and *Eumopsbonariensis*, *E.delticus* can be differentiated from *E.patagonicus* by being larger in some external and cranial measurements, including the lengths of forearm and skull, respectively. Additionally, the extension of the internal keel of the ears, not reaching the anterior antitragus area in *E.patagonicus*, is a discreet characteristic that can help differentiate *E.patagonicus* from *E.delticus* (Díaz et al. 2021). However, Bernardi et al. (2009) identified an individual as *E.patagonicus* with an internal keel reaching the middle region of the antitragus. Concerning differentiation of *E.bonariensis*, cranial morphological characters are provided in the Discussion to aid in distinguishing both species.

#### ﻿ Eumops glaucinus

Taxon classificationAnimaliaChiropteraMolossidae

(J.A. Wagner, 1843)

BF382F93-EBDB-5CAC-A468-F7C6A7492820

##### Summary of captures.

Eight females (CMARF 2123–2130) and 13 males (CMARF 2131–2143).

##### External measurements and weights.

Females: TLB: 142.25 (135.00–150.00), TL: 51.32 (41.46–58.00), LHL: 9.86 (7.91–11.00), EL: 22.33 (21.00–25.00), W: 33.12 (27.00–36.00). Males: TLB: 144.00 (137.00–148.00), TL: 51.84 (45.00–58.00), LHL: 10.53 (9.00–12.00), EL: 23.93 (22.18–26.00), W: 31.46 (28.00–36.00).

##### Morphological description.

**Figure 7.**
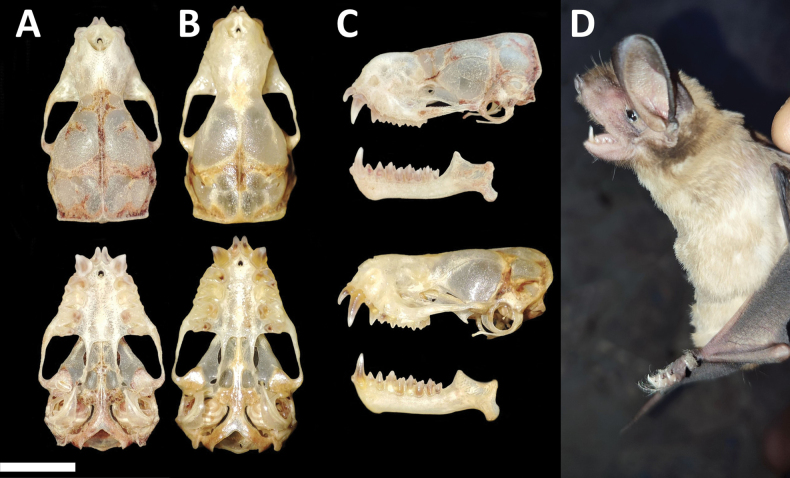
Dorsal, ventral, and lateral views of the skull of a female (CMARF 2127 **A, C** upper) and a male (CMARF 2131 **B, C** lower) of *Eumopsglaucinus* collected in the Caatinga (northeastern Brazil), showing sexual dimorphism in some dimensions **D** external body features of a female of *E.glaucinus* from the same locality (CMARF 2127). Scale bar 5 mm.

The fur color is dark brown to cinnamon, grayish dorsally, and pale brown ventrally, showing slight contrast between both sides (Fig. 7). The ears are wider than long and joined at the forehead. Antitragus is well developed, with a broad base. The tragus is small, wide, and square in shape. Snout elongated, with smooth upper lips. Gular-thoracic gland present in males.

Skull elongated, with variations in the posterior portion of the braincase; in some specimens, the posterior region is more elevated, while in others, it is flatter (Fig. 7). Similarly, the sagittal crest in some specimens is notably well-developed. In contrast, it is less perceptible to others. The lambdoidal crest is developed and evident. The basisphenoid pits are deep. Palate arched. The upper incisors are fused at the bases and divergent at the tips. Some skull measurements are presented in Table 1.

##### Identification.

Externally, the forearm < 65 mm, and the short ears (averaging < 34 mm in both sexes) differentiate this species from the largest members of *Eumops* (*E.chimaera*, Gregorin, Moras, Acosta, Vasconcellos, Poma, dos Santos & Paca, 2016, *E.dabbenei* O. Thomas, 1914, *E.perotis* (Schinz, 1821), and *Eumopstrumbulli* O. Thomas, 1901), while the small, wide, square-shaped tragus, besides the pale brown pelage coloration, separates it from *Eumopsauripendulus* (G. Schaw, 1800), which presents a pointed tragus and a blackish pelage (Eger 2008).

#### ﻿ Molossops temminckii

Taxon classificationAnimaliaChiropteraMolossidae

(Burmeister, 1854)

7EEC5847-1250-5493-8AC1-2AB97B91798C

##### Summary of captures.

One female (CMARF 2144) and one male (CMARF 2145).

##### External measurements and weights.

Female: TLB: 85.00, TL: 33.00, LHL: 5.00, EL: 12.00, W: 6.00. Male: TLB: 80.00, TL: 28.00, LHL: 5.00, EL: 13.00, W: 6.00.

##### Morphological description.

**Figure 8.**
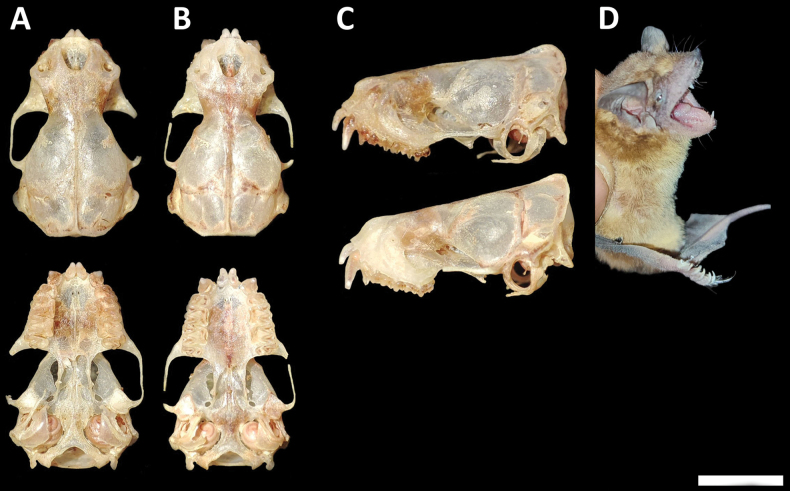
Dorsal, ventral, and lateral views of the skull of a female (CMAR 2144 **A, C** upper) and a male (CMARF 2145 **B, C** lower) of *Molossopstemminckii* collected in the Caatinga, northeastern Brazil **D** external body features of a female of *Molossopstemminckii* from the same locality. Scale bar 5 mm.

Dorsal pelage is chestnut-brown at the tips and yellow at the bases, while ventrally, the coloration is slightly paler (Fig. 8). Ears triangular, with a small and triangular tragus. Antitragus broad and slightly inclined posteriorly. Snout elongated, flat, wide, and blunt, featuring a slightly prominent tip and an obtuse projection between the nasal orifices. Lips smooth and bordered by a fine fringe of hook-shaped hairs, accompanied by a tuft of bristles below the nostrils. Small warts or papillae are notably present on the upper edge of the nostrils.

**Figure 9.**
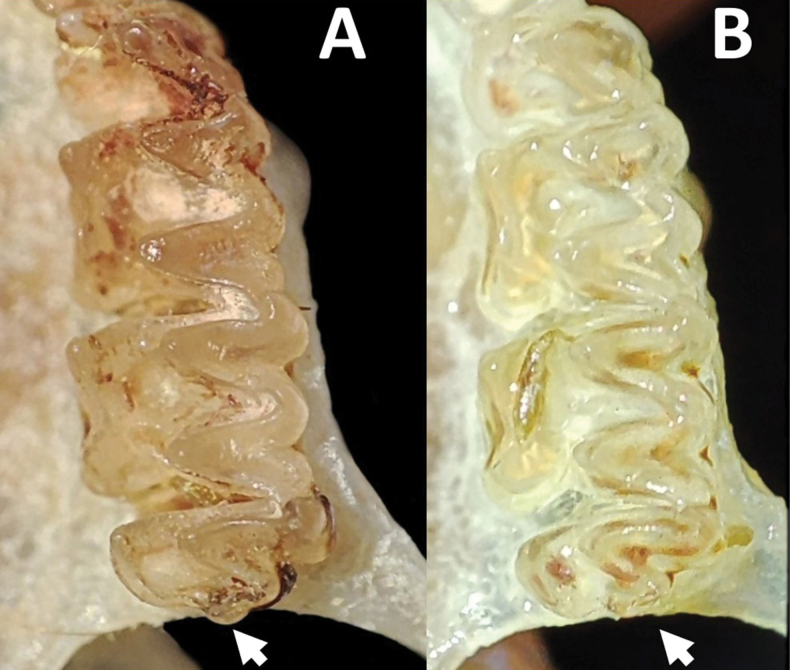
Magnified view (not to scale) of upper molar teeth in specimens of *Molossopstemminckii*, showing the sexual dimorphism in the development of the third molar indicated with white arrows (**A** female **B** male).

Skull with dorsoventral flattening (Fig. 8), characterized by an elevation extending from the nasal tip to the posterior part of the braincase. Postorbital constriction is prominently defined, accompanied by deep lacrimal canals. The sagittal crest is distinctive, reaching its peak at the junction with a well-developed lambdoidal crest. Tympanic bullae small. Basisphenoid pits are shallow. Third upper molar is well-developed in the female, surpassing the maxillary bone (Fig. 9). Third upper molar is less developed in the male, not extending beyond the maxillary bone (Fig. 9). Lower incisors are bilobed. Some skull measurements are shown in Table 1.

##### Identification.

The relatively small size (forearm < 33 mm) and condylobasal length < 15 mm allowed for the assignment of the denomination *M.temminckii* to the specimens referred to here, distinguishing them from the only known congener in Brazil (*M.neglectus* S.L. Williams & Genoways, 1980), whose reported distribution corresponds to the southeastern region (Gregorin et al. 2020). *Molossopsgriseiventer* Sanborn, 1941 a taxon recently validated as a full species (Ramírez-Chaves et al. 2023), has an Andean distribution in Colombia. It can be differentiated from *M.temminckii* by the less pronounced shape of the nasal septum. However, it is important to consider that Ramírez-Chaves et al. (2023) did not provide information on sex differences in their comparisons. The variations between *M.temminckii* and *M.griseiventer*, suggested by Ramírez-Chaves et al. (2023), could be related to sexual dimorphism, as reported in our study. These authors compared both species without considering the marked sexual dimorphism in Molossidae (Eger 2008); future studies could clarify the possible separation between *M.griseiventer* and *M.temminckii*.

###### ﻿*Molossops* sp.

**Summary of captures.** One female (CMARF 2146).

**External measurements and weight.**TLB: 71.00, TL: 21.14, LHL: 4.87, EL: 11.03, W: 6.60.

**Morphological description.** The dorsal pelage is chocolate brown at the tips and yellow at the base, while the coloration is slightly paler ventrally. Ears triangular, with small and triangular tragus. Antitragus broad and slightly inclined posteriorly. Snout elongated, flat, wide, and blunt, featuring a slightly prominent tip and an obtuse projection between the nasal orifices. Lips smooth and bordered by a fine fringe of hook-shaped hairs, accompanied by a tuft of bristles below the nostrils.

The skull exhibits dorsoventral flattening, with a slight elevation from the tip of the nasals to the back of the braincase. Postorbital constriction is prominently defined, accompanied by deep lacrimal canals. The sagittal crest is present but is low at the junction with the lambdoidal crest. Lambdoidal crest weakly developed. Tympanic bullae are small, and the basisphenoid pits are shallow. The third upper molar has two well-developed posterior commissures, surpassing the maxillary bone. Lower incisors are trilobed. Some skull measurements are shown in Table 1.

**Identification.** The presence of three-lobed lower incisors and two well-developed posterior commissures in the third upper molar, surpassing the maxillary, represent unique characteristics that differentiate this specimen from the two specimens previously assigned to *Molossopstemminckii* or its other congener, *Molossopsgriseiventer*.

#### ﻿ Molossus aztecus

Taxon classificationAnimaliaChiropteraMolossidae

Saussure, 1860

CDBAB67F-F677-552A-85A6-3F46383F9206

##### Summary of captures.

Three females (CMARF 2147–2149) and two males (CMARF 2150 and 2151).

##### External measurements and weights.

Females: TLB: 105.00 (103.00–107.00), TL: 40.66 (38.00–44.00), LHL: 6.33 (6.00–7.00), EL: 11.00 (10.00–12.00), W: 11.33 (10.00–12.00). Males: TLB: 110.00 (110.00–110.00), TL: 40.44 (38.89–42.00), LHL: 6.00 (6.00–6.00), EL: 12.30 (12.00–12.60), W: 12.50 (12.00–13.00).

##### Morphological description.

Dorsal pelage with a uniform chocolate brown color and shorter at the shoulders (2–3 mm). Ventral fur is slightly bicolored, with the basal portion of hairs chocolate brown and the tips varying between dark brownish and black shade.

**Figure 10.**
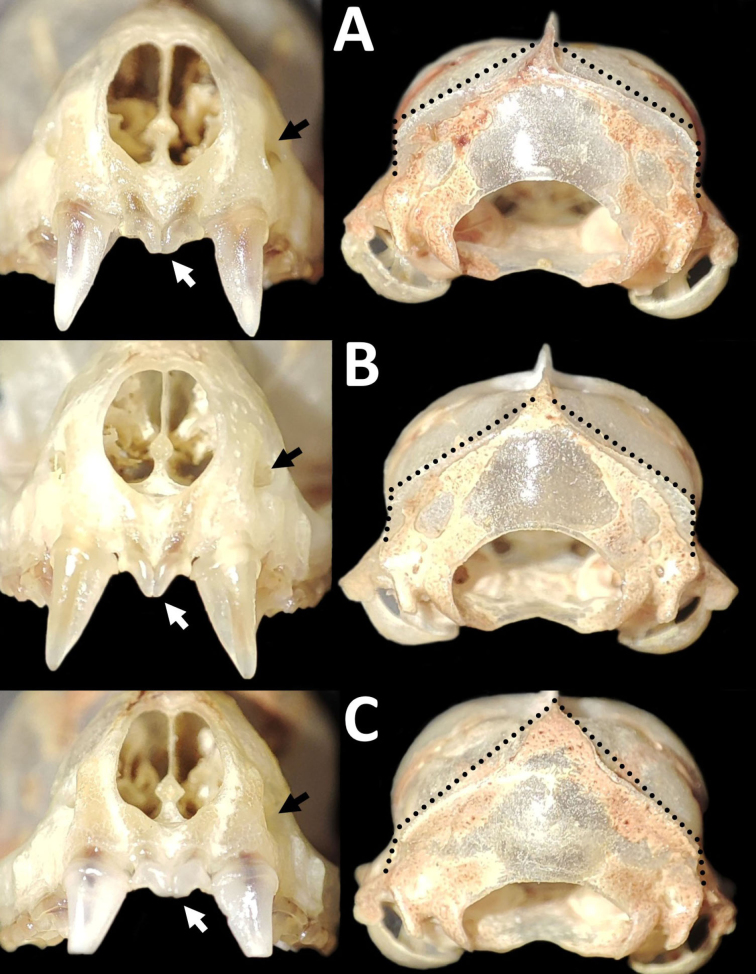
**A***Molossusaztecus* (CMARF 2147) **B***Molossusmolossus* (CMARF 2158) **C***Moloussuscurrentium* (CMARF 2154), collected in the Caatinga (northeastern Brazil), showing differences in the shape of the upper incisors (left), and the occipital complex shape in the posterior region of the skulls (right).

Skull with an inflated rostrum and a rounded braincase. Infraorbital foramen opens laterally in frontal view. Upper incisors spatulated. Occipital complex with a rectangular shape (Fig. 10). Canines projected anteriorly. The nasal process in males is well-developed. Basioccipital pits moderately depth. Lambdoidal and sagittal crests well-developed. Mastoid process oriented ventrally in posterior view. The mandible has a developed angular process and a pair of bilobed incisors. Some skull measurements are shown in Table 2.

**Table 2.** External and cranial measurements for four species of bats of the family Molossidae with confirmed identity and three unidentified morphos. Data based on specimens collected in the Caatinga bioma (Lençóis, Chapada Diamantina region, northeastern Brazil), according to this study (catalog number are indicated) and previous information in literature. Abbreviations are described in the section of materials and methods.

TAXA	FA	GSLI	GSL	CBL	CIL	PL	ZB	MB	BCW	IOW	C-M3	C-C	M3-M3	LM	c-m3
*Molossusaztecus*															
Female (CMARF 2147)	39.75	16.61	15.90	14.56	15.66	5.44	10.90	10.11	8.72	3.77	6.12	4.26	7.75	11.66	7.14
Female (CMARF 2148)	38.79	16.81	15.50	14.93	15.04	5.85	10.40	9.84	8.55	3.38	5.99	4.51	7.94	11.28	6.83
Female (CMARF 2149)	40.76	16.28	15.50	14.99	15.20	5.86	10.57	9.84	8.42	3.42	5.73	4.19	7.63	11.29	6.62
Females (average of Loureiro et al. 2018b)	39.00	16.68	16.41	–	13.37*	5.29	10.65	–	9.02	3.78	6.04	4.32	7.62	–	–
Male (CMARF 2150)	39.46	16.74	16.00	14.90	15.07	6.01	10.28	9.89	8.44	3.35	5.69	4.42	7.90	11.64	6.18
Male (CMARF 2151)	40.74	17.04	16.00	15.86	15.47	6.26	10.68	10.52	8.61	3.69	6.17	4.34	8.02	11.73	7.32
Male (CMUFV 1668, Gregorin et al. 2011)	38.50	–	–	–	16.04	–	–	–	9.50	4.0	6.30	–	7.80	–	6.70
Male (CMUFV 1664, Gregorin et al. 2011)	37.80	–	–	–	16.00	–	–	–	9.30	4.0	6.40	–	8.10	–	6.70
Male (CMUFLA 399, Gregorin et al. 2011)	38.80	–	–	–	16.50	–	–	–	9.60	3.90	6.30	–	8.00	–	6.90
Male (CMUFLA 400, Gregorin et al. 2011)	39.30	–	–	–	16.20	–	–	–	9.40	3.90	6.40	–	8.10	–	6.70
Male (CMUFLA 416, Gregorin et al. 2011)	39.00	–	–	–	16.40	–	–	–	9.60	3.90	6.50	–	8.30	–	6.70
Males (average of Loureiro et al. 2018b)	39.50	17.62	17.28	–	14.01*	5.53	10.91	–	9.10	3.85	6.21	4.66	7.88	–	–
*Molossuscurrentium*															
Female (CMARF 2152)	38.82	15.04	14.98	14.93	14.84	5.82	10.02	10.08	8.38	3.55	5.66	4.21	7.35	11.30	6.64
Female (CMARF 2153)	38.98	17.04	15.58	15.07	14.74	5.20	10.18	10.15	8.72	3.34	5.84	4.32	7.62	11.19	6.46
Female (CMARF 2154)	39.59	16.14	15.60	14.97	14.67	5.65	10.67	10.08	8.34	3.73	5.71	4.35	7.80	11.25	6.52
Female (CMARF 2155)	40.39	16.59	15.78	14.94	14.54	5.87	10.29	10.04	8.62	3.57	5.77	4.16	7.74	11.21	6.76
Female (CMARF 2156)	37.81	16.67	15.75	14.58	14.92	5.67	10.36	10.06	8.70	3.67	5.98	4.23	7.02	11.13	6.15
Females (average of Loureiro et al. 2018b)	41.90	17.97	17.57	–	14.34*	5.54	11.62	–	9.32	4.13	6.61	4.82	8.25	–	–
*Molossusmolossus*															
Female (CMARF 2157)	38.00	15.70	15.00	14.20	14.44	5.95	10.23	10.10	8.44	3.71	5.56	4.07	7.53	11.07	6.55
Female (CMARF 2158)	40.49	16.51	15.46	15.05	15.07	6.03	9.80	10.27	8.43	3.69	5.84	4.52	7.49	11.72	6.65
Female (CMARF 2159)	39.82	16.42	15.56	14.97	15.07	6.06	10.32	10.03	8.55	3.60	6.04	4.47	7.80	11.41	6.87
Female (CMARF 2160)	38.43	16.83	15.98	15.20	15.55	5.81	10.22	9.94	8.64	3.57	5.89	4.36	7.69	11.33	6.69
Female (CMARF 2161)	40.93	16.98	15.99	15.43	15.47	5.78	10.47	10.51	8.58	3.58	6.12	4.40	7.93	11.84	6.82
Female (CMARF 2162)	38.97	16.39	15.98	14.98	15.06	5.53	10.71	10.43	8.45	3.76	6.00	4.30	7.83	11.44	6.73
Female (CMARF 2163)	39.45	16.54	15.78	15.04	15.10	5.50	10.71	10.54	8.66	3.64	5.81	4.32	7.92	11.26	6.76
Female (CMARF 2164)	38.23	16.11	15.78	15.03	15.20	5.87	10.17	10.06	8.35	3.67	6.31	4.03	7.62	11.40	6.75
Female (CMARF 2165)	40.30	16.30	15.95	14.99	15.05	5.63	10.24	10.04	8.37	3.60	5.94	4.10	7.60	11.31	6.79
Female (CMARF 2166)	38.23	16.29	15.78	14.57	14.63	5.27	10.16	10.13	8.37	3.54	5.88	4.15	7.82	11.21	6.97
Female (CMARF 2167)	39.19	16.06	15.65	14.60	14.86	5.91	10.25	10.08	8.78	3.52	5.99	4.12	7.64	11.41	6.67
Female (CMARF 2168)	39.51	16.27	15.78	14.85	14.87	5.42	10.30	10.06	8.38	3.62	5.88	4.18	7.96	11.21	6.83
Female (CMARF 2169)	39.02	16.24	15.50	13.99	14.55	5.41	10.11	9.90	8.79	3.65	5.91	4.34	7.53	11.26	6.77
Female (CMARF 2170)	40.57	16.08	15.98	14.44	14.86	5.18	10.33	9.91	8.57	3.54	5.87	4.26	7.45	11.75	6.89
Females (average of Loureiro et al. 2018b)	39.45	16.95	16.49	–	13.49*	5.39	10.26	–	8.84	3.70	6.13	4.36	7.65	–	–
Male (CMARF 2171)	39.28	16.72	16.00	15.19	15.21	5.98	10.65	10.41	8.88	3.86	6.10	4.27	8.02	11.22	6.73
Males (average of Loureiro et al. 2018b)	40.21	17.65	17.22	–	14.04*	5.54	11.02	–	9.05	3.80	6.30	4.58	7.92	–	–
*Molossusrufus*															
Female (CMARF 2172)	50.75	21.49	21.00	19.15	19.72	7.02	13.26	13.32	10.61	4.38	8.00	5.72	10.03	15.56	8.89
Female (CMARF 2173)	50.16	20.32	19.10	17.92	18.92	7.48	13.34	12.88	10.65	4.55	7.73	5.73	9.95	15.20	8.81
Female (CMARF 2174)	50.76	21.27	19.00	18.24	18.86	6.62	13.06	12.34	10.42	4.29	7.78	5.49	9.84	14.69	8.46
Female (CMARF 2175)	50.41	20.67	19.10	18.79	19.48	6.66	13.62	13.55	10.57	4.39	7.69	5.69	10.14	14.93	8.67
Female (CMARF 2176)	50.94	20.86	19.00	18.34	18.75	6.73	13.40	13.01	10.59	4.41	7.69	5.69	10.12	14.84	9.09
Female (CMARF 2177)	49.25	20.37	19.10	17.84	18.57	7.31	13.37	13.25	10.76	4.06	7.67	5.55	9.96	14.87	8.87
Female (CMARF 2178)	50.73	21.54	19.57	18.61	19.38	6.89	13.35	12.62	10.46	4.37	7.64	5.51	9.81	14.51	8.59
Female (CMARF 2179)	50.03	21.95	21.10	19.15	19.93	7.19	13.32	13.11	10.07	4.98	7.93	6.23	9.95	15.29	8.93
Female (CMARF 2180)	50.67	20.68	19.56	18.23	19.22	6.79	13.22	12.62	10.43	4.39	7.48	5.56	10.10	14.81	8.72
Female (CMARF 2181)	49.94	20.58	19.98	18.13	19.25	6.80	13.25	12.70	10.44	4.35	7.50	5.55	10.15	14.71	8.12
Females (average of Loureiro et al. 2018b)	50.00	21.30	20.69	–	17.07*	6.78	12.90	–	10.62	4.38	7.74	5.58	9.67	–	–
Male (CMARF 2182)	51.03	22.57	21.99	19.82	20.51	7.03	14.28	13.61	10.99	4.88	8.03	6.08	10.21	15.86	9.19
Male (CMARF 2183)	52.26	21.71	21.00	19.99	20.69	7.42	13.85	13.99	10.69	4.36	7.79	6.03	10.11	16.03	8.26
Male (CMARF 2184)	52.42	22.20	21.98	19.98	20.58	7.08	14.45	14.30	10.70	4.53	8.12	6.04	10.69	16.18	9.26
Male (CMARF 2185)	50.59	23.26	21.98	19.88	20.66	6.92	14.37	13.79	10.86	4.49	8.29	6.32	10.48	16.17	9.47
Male (CMARF 2186)	50.98	21.68	21.00	18.89	19.73	7.67	13.43	13.72	10.51	4.58	7.79	5.94	10.28	15.43	9.02
Male (CMARF 2187)	51.12	22.87	21.56	20.02	20.60	6.84	13.86	14.00	10.68	4.41	7.93	6.30	10.40	15.89	9.16
Male (CMARF 2188)	52.55	22.84	21.50	20.17	20.85	7.98	14.38	14.22	11.16	4.71	8.02	6.19	10.62	16.08	9.68
Male (CMARF 2189)	51.14	22.41	21.78	19.96	20.67	7.42	14.72	14.00	11.09	4.80	8.28	6.33	10.56	16.09	9.34
Male (CMARF 2190)	53.21	22.75	21.54	19.40	20.56	8.00	14.25	13.87	10.68	4.62	7.93	6.07	10.20	16.27	9.23
Male (CMARF 2191)	53.14	23.04	21.00	19.51	20.28	7.24	14.63	14.22	11.08	4.74	8.10	6.61	10.34	16.57	9.10
Male (CMARF 2192)	53.14	22.04	21.15	19.56	19.86	7.15	13.62	13.44	11.15	4.59	7.88	5.66	10.38	15.69	9.17
Male (CMARF 2193)	50.38	22.38	21.00	19.47	20.19	7.34	14.29	14.18	10.79	4.53	7.75	6.06	10.38	15.65	9.05
Male (CMARF 2194)	52.37	22.36	21.45	19.46	20.40	7.70	14.06	13.75	10.75	4.41	7.89	5.94	10.30	15.41	8.98
Male (CMARF 2195)	53.42	22.82	21.20	19.46	20.20	7.19	14.02	13.74	10.70	4.64	7.97	6.24	10.51	15.77	9.29
Male (CMARF 2196)	45.83	21.50	21.00	19.00	19.46	6.73	14.26	13.65	10.66	4.29	7.71	6.17	10.40	15.60	9.06
Male (CMARF 2197)	45.83	21.55	21.00	19.00	19.00	6.50	14.25	13.80	10.50	4.69	7.90	6.07	10.51	15.70	9.20
Males (average of Loureiro et al. 2018b)	49.55	22.9	22.19	–	17.85*	7.09	14.05	–	10.91	4.54	8.04	6.07	9.95	–	–
*Molossus* sp. 1															
Female (CMARF 2198)	39.19	16.48	15.96	14.79	15.02	5.77	10.51	10.26	8.78	3.70	5.65	4.09	7.66	11.17	6.58
Male (CMARF 2199)	40.53	17.56	16.00	15.50	15.55	5.93	10.76	10.41	8.45	3.66	5.94	4.36	8.11	11.65	6.49
Male (CMARF 2200)	41.02	17.31	16.00	15.12	15.20	6.01	10.57	10.70	8.79	3.67	6.15	4.34	7.89	11.47	6.81
Male (CMARF 2201)	41.70	17.15	16.16	14.99	15.51	5.63	10.54	10.21	8.46	3.53	6.05	4.49	8.00	11.75	7.00
Male (CMARF 2202)	38.96	16.00	15.78	15.14	15.37	5.85	10.15	9.69	8.35	3.67	5.92	4.09	7.62	11.41	6.92
Male (CMARF 2203)	40.19	17.15	16.55	15.88	16.20	6.10	11.06	10.61	8.81	3.81	5.99	4.60	7.98	11.91	6.98
Male (CMARF 2204)	40.48	17.69	16.76	16.06	16.08	6.03	11.03	10.61	8.80	4.00	6.19	4.49	8.10	12.15	7.34
Male (CMARF 2205)	38.47	17.12	16.19	15.48	15.32	5.41	10.37	10.00	9.48	3.81	5.95	4.39	7.56	12.11	6.76
*Molossus* sp. 2															
Male (CMARF 2206)	40.16	16.61	16.00	15.28	15.50	6.18	10.70	10.75	8.62	3.80	6.00	4.45	7.71	11.54	6.66
Male (CMARF 2207)	39.58	17.56	16.17	15.17	15.27	5.34	10.83	10.59	8.59	3.72	6.06	4.34	8.07	11.39	7.67
Male (CMARF 2208)	39.05	16.00	15.96	14.49	14.69	5.00	10.29	9.78	8.53	3.45	5.87	4.05	7.53	10.77	6.53
*Molossus* sp. 3															
Female (CMARF 2209)	40.26	16.15	16.00	15.21	15.90	5.09	10.08	10.25	8.58	3.51	5.89	4.58	7.63	11.43	6.65

*This variable was incorrectly measured: Loureiro et al. (2018b) measured from the base of the tympanic bullae and not from the base of the condyle.

##### Identification.

The unicolored dorsal pelage, the infraorbital foramen opens laterally in frontal view, the upper incisors spatulated, and the rectangular-shaped occipital complex differentiate the individuals of this species from their morphologically closest related congeners: *Molossuscoibensis* J.A. Allen, 1904, *M.currentium* O. Thomas, 1901, and *M.molossus* (Pallas, 1766) (Gregorin et al. 2011; Loureiro et al. 2018a, 2018b).

#### ﻿ Molossus currentium

Taxon classificationAnimaliaChiropteraMolossidae

O. Thomas, 1901

80DCF7A3-B221-52FC-B76A-7519DFE031C0

##### Summary of captures.

Five females (CMARF 2152–2156).

##### External measurements and weights.

TLB: 103.20 (102.00–105.00), TL: 42.80 (42.00–45.00), LHL: 6.60 (5.00–7.00), EL: 12.00 (11.00–12.00), W: 10.20 (10.00–11.00).

##### Morphological description.

Dorsal fur coloration shows the following variations: unicolored chocolate brown in two specimens, slightly bicolored in two (hairs with a short pale brown basal band and a broader chocolate brown portion), and notably bicolored in one (with a pale brown base and chocolate brown tips); in all these cases, the dorsal coloration slightly contrasts with the venter. Length of dorsal hairs at the shoulders with the following variations: 4 mm in two specimens, 2 mm in one, and 3 mm in two.

Skull with elongated rostrum and a rounded braincase. Upper incisors spatulate. Infraorbital foramen varies in position: laterally positioned and slightly expanded in two specimens (making it visible in frontal view). In contrast, it is laterally positioned in the other three but not expanded. Occipital complex with a triangular shape (Fig. 10). Canines anteriorly projected in lateral view. The nasal process developed in three specimens and undeveloped in the rest. Basioccipital pits are present but shallow in two specimens and absent in the remaining three. The mastoid process is long and oriented ventrally. Presence of two bilobed lower incisors. Some skull measurements are shown in Table 2.

##### Identification.

The upper incisors are spatulate, the infraorbital foramen is laterally positioned, not expanded, or slightly expanded, and the occipital complex is triangular. These features differentiate the individuals of this species from their morphologically closest related congeners: *Molossusaztecus* Saussure, 1860, and *M.molossus* (Gregorin et al. 2011; Loureiro et al. 2018a, 2018b).

#### ﻿ Molossus molossus

Taxon classificationAnimaliaChiropteraMolossidae

(Pallas, 1766)

9DFDE743-B587-5141-B4C8-E19F6E02940A

##### Summary of captures.

13 females (CMARF 2157–2169) and one male (CMARF 2171).

##### External measurements and weights.

Females: TLB: 102.38 (100.00–115.00), TL: 40.68 (36.00–45.00), LHL: 6.68 (5.95–7.88), EL: 11.92 (10.00–16.00), W: 11.30 (10.00–13.00). Male: TLB: 105.00, TL: 30.00, LHL: 6.68, EL: 12.70, W: 12.00.

##### Morphological description.

**Figure 11.**
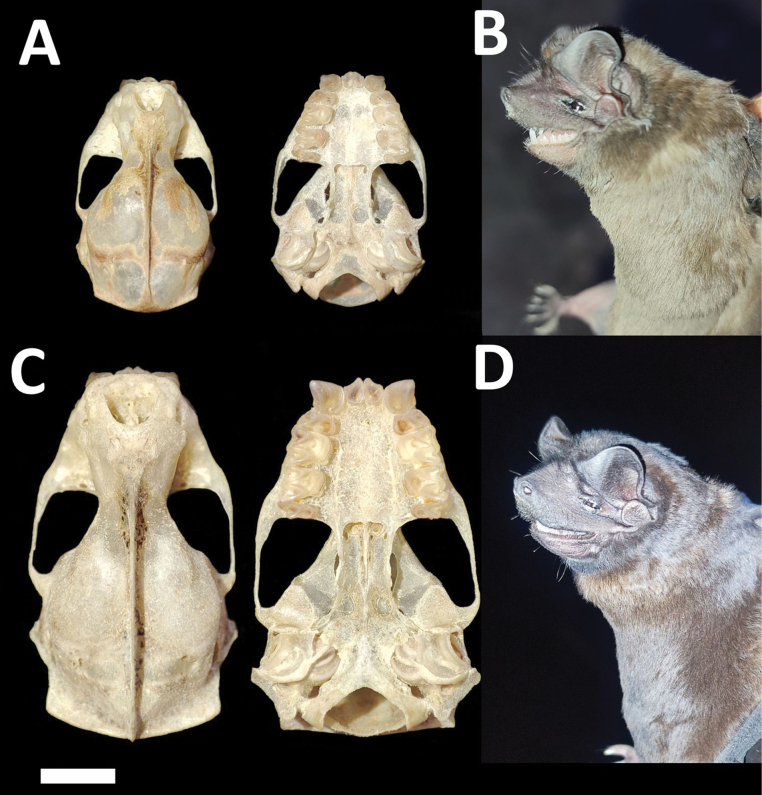
**A, B***Molossusmolossus* (CMARF 2159) and **C, D***Molossusrufus* (CMARF 2182) **A, C** dorsal and ventral views of the skull in two species of *Molossus* collected in the Caatinga, northeastern Brazil **B, D** individuals of *M.molossus* and *M.rufus* photographed in the field (not to scale). Scale bar 5 mm.

Rostrum narrow with a developed keel (Fig. 11). Dorsal hairs are notably bicolored in some females, with broad white bases and dark brown or chocolate hues tips (Fig. 11). Dorsal pelage is slightly bicolored in the male, with a pale brown base and a broader dark brown or chocolate hues distal band. Ventral coloration is paler brown, subtly contrasting the dorsal side in all specimens. Length of dorsal hairs at the shoulders: 2–3 mm in females and 3 mm in the male.

Upper incisors with parallel tips, projecting anteriorly from the rostrum. Infraorbital foramen anteriorly positioned. Nasal process present. The occipital complex is triangular (Fig. 10). Basioccipital pits are absent in some females and in the male, while they are present but barely visible in other females. Sagittal and lambdoidal crests are present but with different degrees of development, showing the maximum degree in the male. The mastoid process developed and oriented ventrally. Presence of a pair of bilobed lower incisors. Some skull measurements are shown in Table 2.

##### Identification.

The rostrum is narrow with a developed keel, the dorsal hairs notably bicolored to slightly bicolored, the infraorbital foramen anteriorly positioned, and the occipital complex triangular in shape, differentiate the individuals of this species from their morphologically closest related congeners: *Molossusaztecus* and *M.currentium* (Gregorin et al. 2011; Loureiro et al. 2018a, 2018b).

#### ﻿ Molossus rufus

Taxon classificationAnimaliaChiropteraMolossidae

E. Geoffroy St.-Hilaire, 1805

55CE3C92-A13E-5A4F-ABF0-EFCB4F9CE543

##### Summary of captures.

Nine females (CMARF 2172–2181) and 16 males (CMARF 2182–2197).

##### External measurements and weights.

Females: TLB: 130.09 (120.00–140.00), TL: 47.13 (38.45–50.00), LHL: 9.16 (6.28–11.00), EL: 14.96 (13.00–16.73), W: 28.60 (25.00–35.00). Males: TLB: 137.75 (130.00–145.00), TL: 48.48 (39.43–58.00), LHL: 8.74 (6.59–9.96), EL: 15.68 (14.69–18.02), W: 30.75 (25.00–38.00).

##### Morphological description.

Dorsal and ventral pelage with dark coloration, with predominantly dusky hue unicolored hairs (Fig. 11). Dorsal hairs longer than 4 mm. Skull with an inflated rostrum and an elongated braincase (Fig. 11). Mastoid process extending laterally in posterior view. The occipital region has a square shape. Lambdoidal crests are highly developed and inclined. Sagittal crests are present and well-developed, with a more pronounced development in males. Infraorbital foramen opens laterally in frontal view. Basioccipital pits moderately depth. Rostrum with triangular shape in frontal view. Upper incisors are short and spatulated, with convergent tips. Presence of a pair of bilobed lower incisors. Some skull measurements are shown in Table 2.

##### Identification.

The relatively larger forearm length and skulls, along with the unicolored dorsal and ventral pelage, serve as characteristics to distinguish individuals of *M.rufus* from its smaller congeners (Loureiro et al. 2018b). The short and spatulated upper incisors, with convergent tips, differentiate this species from *Molossuspretiosus* Miller, 1902 (Loureiro et al. 2018b). The length of hairs longer than 4 mm, average forearm lengths of 51.98 mm in males and 50.36 mm in females, along with the greater skull lengths for both sexes of *M.rufus*, distinguish this species from *M.fluminensis* (Loureiro et al. 2020). Furthermore, a pair of lower incisors and other externally visible characters distinguish *M.rufus* from members of the genus *Promops* P. Gervais, 1856 (Eger 2008), which can be confused due to their morphological similarities.

###### ﻿*Molossus* sp. 1

**Summary of captures.** One female (CMARF 2198) and seven males (CMARF 2199–2205).

**External measurements and weights.** Female: TLB: 103.00, TL: 40.00, LHL: 9.00, EL: 12.00, W: 10.00. Males: TLB: 104.00 (98.00–111.00), TL: 39.85 (35.00–46.00), LHL: 6.55 (5.66–7.52), EL: 12.07 (10.00–15.40), W: 13.00 (12.00–14.00).

**Morphological description.** Dorsal fur is unicolored (dark brown), with hairs at the shoulders measuring 2 mm in length. Ventral coloration subtly contrasts with the dorsum, showing pale brown bases. Rostrum broad and convex, with a developed keel. Upper incisors elongated (similar to *M.molossus*) and anteriorly projected. The infraorbital foramen is exposed anteriorly in the female, while in some males, this structure is anteriorly exposed, and in others, it is laterally positioned. Nasal process present. Occipital complex with a triangular shape. Mastoid processes developed (large) and ventrally oriented. Presence of a pair of bilobed lower incisors. Some skull measurements are shown in Table 2.

**Identification.** The morphology of these specimens exhibits unique characteristics in the facial morphology (rostrum broad and convex, with a developed keel), distinguishing them from *M.aztecus*, *M.currentium*, and *M.molossus*. Furthermore, the mastoid processes are developed (large) and oriented ventrally, differentiating them from *Molossus* sp. 2, while the uniform coloration of the dorsal pelage (unicolored) and the broad, convex rostrum with a developed keel, distinguish them from *Molossus* sp. 3.

###### ﻿*Molossus* sp. 2

**Summary of captures**. Three males (CMARF 2206–2208).

**External measurements and weights.**TLB: 102.66 (100.00–106.00), TL: 35.00 (32.00–37.00), LHL: 6.14 (5.23–7.00), EL: 12.28 (11.91–12.95), W: 13.00 (12.00–14.00).

**Morphological description.** Rostrum broad and convex, with a developed keel. Dorsal fur is weakly bicolored (hairs with pale brown bases and a broad band of dark chocolate brown in the rest). Hairs at the shoulders 2 mm in length. Ventral and dorsal coloration with slight contrast shows pale brown bases and chocolate brown tips.

Infraorbital foramen laterally positioned in two specimens and anteriorly positioned in one. Upper incisors elongated (similar to *M.molossus*) and projected anteriorly. Nasal process undeveloped. Basioccipital pits are absent in one, while in two specimens they are present but scarcely visible. Occipital complex with triangular shape. The mastoid process is scarcely developed (short) and ventrally oriented. Presence of a pair of bilobed lower incisors. Some skull measurements are shown in Table 2.

**Identification.** Like *Molossus* sp. 1 (see above), the morphology of these specimens exhibits unique characteristics in facial morphology (broad and convex rostrum with a developed keel), distinguishing them from *M.aztecus*, *M.currentium*, and *M.molossus*. However, the scarcely developed (short) and ventrally oriented mastoid processes set them apart from *Molossus* sp. 1. Additionally, the uniform, weakly bicolored dorsal pelage, combined with the broad and convex rostrum with a developed keel, differentiates them from *Molossus* sp. 3.

###### ﻿*Molossus* sp. 3

**Summary of captures**. One female (CMARF 2209).

**External measurements and weight.**TLB: 101.00, TL: 41.00, LHL: 6.00, EL: 11.00, W: 10.50.

**Morphological description.** Rostrum narrow, with a slightly undeveloped keel. Dorsal fur is bicolored, with a white basal band and dark chocolate brown color in the rest. Ventral fur contrasts with the dorsum, with hairs showing broad gray bases and pale brown tips. Hairs at the shoulders is 3 mm in length. Upper incisors show only the bases (completely worn). Frontal foramen laterally positioned. The nasal process developed. Occipital complex with triangular shape. Mastoid process elongated and ventrally oriented. Basioccipital pits are present but shallow. Presence of a pair of bilobed lower incisors. Some skull measurements are shown in Table 2.

**Identification.** This specimen shows unique characteristics in its facial morphology (narrow rostrum, with poorly developed keel) and in the coloration of the ventral pelage, which contrasts with the dorsum. This morphological and chromatic pattern distinguishes it from other small species of *Molossus* (e.g., *M.aztecus*, *M.currentium*, *M.molossus*, *Molossus* sp. 1, and *Molossus* sp. 2).

#### ﻿ Neoplatymops mattogrossensis

Taxon classificationAnimaliaChiropteraMolossidae

(Vieira, 1942)

3E3C251E-0442-5AF7-BAB6-AB5645C04925

##### Summary of captures.

Seven females (CMARF 2210–2216).

##### External measurements and weights.

TLB: 78.85 (73.00–82.00), TL: 29.16 (25.14–32.00), LHL: 5.95 (5.00–7.00), EL: 12.65 (10.00–13.95), W: 5.37 (5.00–6.60).

##### Morphological description.

**Figure 12.**
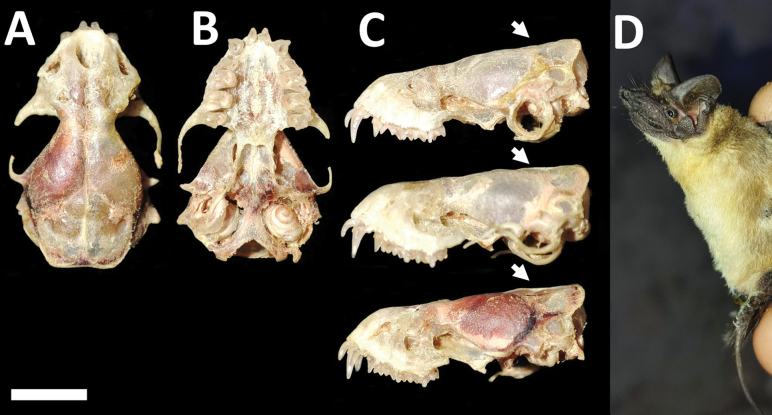
*Neoplatymopsmattogrossensis* (CMARF 2212 **D**) collected in the Caatinga (northeastern Brazil) **A, B** dorsal and ventral views of the skull in a female, in addition to lateral views **C** in three females (from the same locality) with different levels of development of the sagittal crest (indicated by the white arrow): absent (above); present but less developed and of lesser extent (medium); and present, more developed and covering a greater extent on the posterior portion of the skull (below). Scale bar 5 mm.

Small granulations on the dorsal surface of the forearm (wart-like granular structures that represent a distinctive generic characteristic). Dorsal fur is pale brown, contrasting with the ventral coloration composed of hairs with yellowish brown tips and whitish bases. The head is dark brown, with the ears distinctly separated on the forehead (Fig. 12). There is a presence of long and conspicuous vibrissae at the bases of the fingers.

Skull flattened (Fig. 12). The sagittal crest is barely visible with magnification only in two specimens; in the remaining five, this structure is absent (Fig. 12). Lambdoidal crests are present and visible. Upper incisors project anteriorly, separated from each other and the canines. First upper and lower premolars are smaller than the other two homologous teeth. The slender mandible shows a high and triangular coronoid process with a rounded apex and a low condyle. Lower incisors are deeply bifid in six specimens and barely bifid in only one case. Some skull measurements are shown in Table 1.

##### Identification.

The short forearm, the presence of granulations on the dorsal surface of the forearm, and the skull, which is flattened and relatively small, are diagnostic characteristics for individuals of this species (Willig and Jones 1985).

#### ﻿ Nyctinomops laticaudatus

Taxon classificationAnimaliaChiropteraMolossidae

(E. Geoffroy St.-Hilaire, 1805)

8C5BE4C9-C9A6-5DFF-963C-25F39C46E17C

##### Summary of captures.

Six females (CMARF 2217–2222) and two males (CMARF 2223, 2224).

##### External measurements and weights.

Females: TLB: 107.15 (100.00–110.87), TL: 46.86 (41.00–50.17), LHL: 8.30 (7.00–10.00), EL: 14.42 (15.56–19.00), W: 11.66 (9.00–15.00). Males: TLB: 106.00 (105.00–107.00), TL: 49.00 (48.00–50.00), LHL: 7.00 (7.00–7.00), EL: 17.50 (17.00–18.00), W: 10.00 (9.00–11.00).

##### Morphological description.

**Figure 13.**
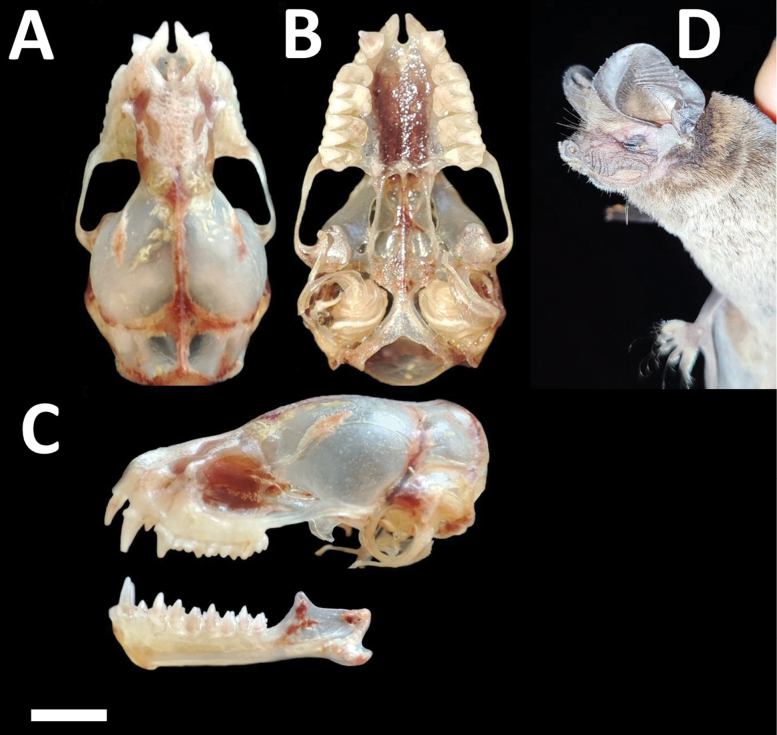
Female of *Nyctinomopslaticaudatus* (CMARF 2217; **D**) collected in the Caatinga, northeastern Brazil **A** dorsal **B** ventral, and **C** lateral views of the skull. Scale bar 5 mm.

The dorsal pelage is dark brown chocolate, with a paler belly (Fig. 13). The ears are relatively long and rounded, with the upper edges converging the forehead. The tragus is small and square, and the antitragus is well-developed and wider at the base. The muzzle is pointed and upturned. Upper lips are deeply furrowed, with vertical folds that overhang the lower lips. Nostrils raised with rows of rectangular papillae on the edge, forming a crest on both sides.

Skull robust (Fig. 13), with sagittal and lambdoidal crests present but not well-developed (only discernible under magnification). Basisphenoid pits deep. Anterior border of palate emarginated. Premaxillaries scarcely separated at the anterior portion. The upper incisors are barely parallel. Lacrimal processes are well-developed. The first lower premolar is in contact with the canine, and the larger second premolar. Third lower molar is diminutive, with a complete commissure. Some skull measurements are shown in Table 1.

##### Identification.

The forearm length < 47 mm, the greatest length of the skull < 19 mm, and the shallow basisphenoid pits distinguish individuals of this species from its congeners, *N.macrotis* (Gray, 1839) and *N.aurispinosus* (T. R. Peale, 1848) (Eger 2008).

#### ﻿ Nyctinomops macrotis

Taxon classificationAnimaliaChiropteraMolossidae

(Gray, 1839)

DC2E867B-D8F3-52FB-BCAD-AA653492BB48

##### Summary of captures.

One female (CMARF 2225).

##### External measurements and weight.

TLB: 137.00, TL: 60.00, LHL: 7.00, EL: 27.00, W: 22.00.

##### Morphological description.

**Figure 14.**
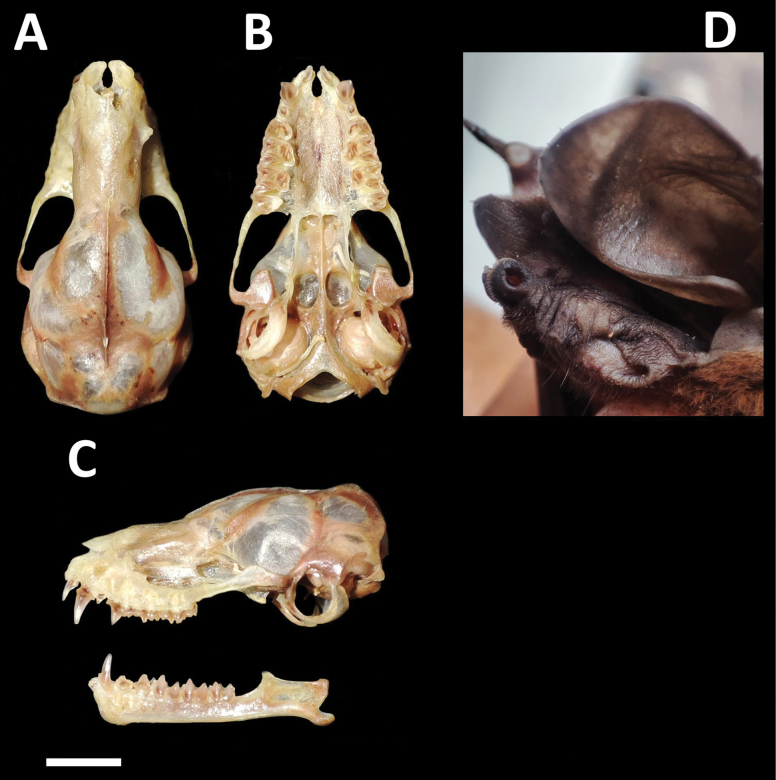
Female of *Nyctinomopsmacrotis* (CMARF 2225; **D**) collected in the Caatinga, northeastern Brazil **A** dorsal **B** ventral, and **C** lateral views of the skull. Scale bar 5 mm.

Dorsal and ventral pelage reddish brown. Ears large, fused at the midline of the forehead and nearly reaching the nostrils. Nostrils directed laterally. The upper lip is deeply furrowed by wrinkles (Fig. 14).

The skull is large, with a narrow rostrum (Fig. 14). Sagittal and lambdoidal crests are present and prominent. Upper incisors parallel. First upper premolar diminutive. Lacrimal processes are well-developed. Basisphenoid pits are large and deep. Lower incisors bifid. Some skull measurements are shown in Table 1.

##### Identification.

The forearm length is > 55 mm, the total length of the skull is > 22 mm, and the basisphenoid pits are large and relatively deep, distinguishing the only captured individual of this species from *N.laticaudatus* and *N.aurispinosus* (Eger 2008).

## ﻿Discussion

Our results document the confirmed presence of at least 12 species from the family Molossidae in the Caatinga biome: *Cynomopsplanirostris*, *Eumopsbonariensis*, *Eumopsdelticus*, *Eumopsglaucinus*, *Molossopstemminckii*, *Molossusaztecus*, *Molossuscurrentium*, *Molossusmolossus*, *Molossusrufus*, *Neoplatymopsmattogrossensis*, *Nyctinomopslaticaudatus*, and *Nyctinomopsmacrotis*. Among them, nine were known for this region based on voucher specimens (Loureiro et al. 2018b; Silva et al. 2018), two (*Eumopsbonariensis* and *Molossuscurrentium*) had been reported informally without conclusive evidence (Silva et al. 2018), and one (*Eumopsdelticus*) represents the first record for the Caatinga. Four additional specimens identified as morphospecies belonging to the genera *Molossops* (one) and *Molossus* (three) were only identified at the genus level, with species-level identification requiring further detailed morphological and molecular analysis.

The morphological patterns and measurements of those specimens representative of *Cynomopsplanirostris*, *Eumopsglaucinus*, *Molossusrufus*, *Nyctinomopslaticaudatus*, *N.macrotis*, *Neoplatymopsmattogrossensis*, *Molossopstemminckii*, *Molossusaztecus*, and *Molossusmolossus*, align with previous data reported within their global distributions (Willig and Jones 1985; Milner et al. 1990; Best et al. 1997; Avila-Flores et al. 2002; Gregorin et al. 2011; Souza et al. 2016; Loureiro et al. 2018b; Gamboa Alurralde and Díaz 2019; López Berrizbeitia and Díaz 2021) and the Caatinga biome (Gurgel-Filho et al. 2015; Rocha et al. 2015; Souza et al. 2016). However, the length of dorsal hairs in specimens of *Molossusaztecus* collected in this study is relatively short. It does not exceed 3 mm, contrary to the morphological diagnosis presented for the species in other areas of the Caatinga (Ceará, Piauí, and Pernambuco) and non-Caatinga regions, where individuals of this species are characterized by long fur reaching 6 mm in length (Gregorin et al. 2011; Loureiro et al. 2018b). In the case of *N.mattogrossensis*, the ear lengths in females collected during our study do not correspond to the values reported by Gurgel-Filho et al. (2015) in Ceará. These authors indicated an average ear length of 19 mm for three individuals, in contrast to our average of 12.65 mm for seven specimens; this last value corresponds to the range reported by Willig and Jones (1985) for females of this species.

The presence of *C.planirostris* in the Caatinga, based on voucher material, has been confirmed in Ceará (Fabián 2008; Novaes and Laurindo 2014; Gurgel-Filho et al. 2015; Feijó and da Rocha 2017), Minas Gerais (Oliveira et al. 2003; Oliveira 2004; Nogueira et al. 2015), Paraíba (Feijó and Langguth 2011; Leal et al. 2013; Beltrão et al. 2015), Pernambuco (Mares et al. 1981; Oliveira et al. 2003; Oliveira 2004; Feijó and Langguth 2011), and Piauí (Novaes et al. 2013); the specimens reported here would be the first records of this molossid for the Bahian Caatinga.

In the case of *Eumopsglaucinus*, information on the geographic distribution in Brazil was recently updated based on a new record in the Maranhão state within the Cerrado biome (Souza Cardoso et al. 2022). These authors do not document the presence of this species in the Caatinga; however, there are previous published records for this biome, based on museum specimens (Astúa and Guerra 2008; Feijó and Langguth 2011) and observations at roosting sites (Vilar et al. 2016). The specimens reported here confirm the presence of *Eumopsglaucinus* in the Bahian Caatinga and represent the second record for the state of Bahia (Sá-Neto and Marinho-Filho 2013).

Both *Eumopsbonariensis* and *Molossuscurrentium* were previously reported in the Caatinga by Silva et al. (2018); however, these authors did not include morphological characters or other evidence to corroborate the identifications, nor did they provide details on the voucher specimens. Additionally, two previous studies conducted acoustic sampling that complemented the capture data (Silva and Bernard 2017; Leal et al. 2022), recording *Molossus* cf. *currentium*. This record suggests that the acoustic identification is believed to be similar to that of *Molossuscurrentium*, although it could not be confirmed. Due to their high flight capacity and the difficulty in capturing them for identification, it is estimated that nearly 75% of New World molossid species remain acoustically unverified (Miller, pers. comm.), including *M.currentium* among them. This is further complicated by early published accounts of hand-released bats and recordings at roosts, especially for molossids, as it is now understood that they usually do not emit diagnostic search phase calls useful for free flight identification, but “clutter” calls typically recorded when entering or departing roosts.

For example, O’Farrell and Miller (1999) described the calls of *M.alvarezi* (then recognized as *M.sinaloae*) characterized as having pulses in triplets vs. the paired calls of *M.molossus* and *M.nigricans*. Subsequently, such triplets emitted by species of *Molossus* are now known to be typical “clutter calls” emitted as bats enter or leave roosts and not the typically paired diagnostic search phase pulses emitted when free-flying. This also has a bearing on other published molossid call parameters that included triple pulses vs. diagnostic paired-pulse for species of *Molossus*, as reported by Jung et al. (2014).

The uncertainty of acoustic identification may result from various factors, including a small sample size, the lack of verified vocal signatures, an understanding of vocal repertoires of a given species, or the call variation seen under varied recording conditions, e.g., hand releases, roost, or enclosure recordings (Jung et al. 2014; Miller et al. 2023). Given that many species of Molossidae are cryptic or highly variable in their morphology this may result in uncertainty of identification even with bats in hand or, in some cases, may be based on older taxonomy along with a lack of comprehensive confirmed vocal signatures matching known species (Jung et al. 2014; Eger 2008; Loureiro et al. 2018b; Miller et al. 2023). Therefore, we suggest that the previous records of *E.bonariensis* and *M.currentium* are not sufficiently supported to be accepted.

The average measurements reported by Loureiro et al. (2018b) for both sexes of *M.currentium* in Brazil, Colombia, and Panama are greater than those of the specimens from the Caatinga. This species was previously reported in Brazil by a single individual collected at Corumbá, Matto Grosso do Sul (Loureiro et al. 2018b). A second record was subsequently published from the Carlos Botelho State Park (São Paulo State) by Cláudio et al. (2020). Our record is the first occurrence in the Caatinga based on voucher specimens, expanding its distribution 1,825 km SE from Corumbá to Lençóis and 1,487 km SE from Carlos Botelho State Park to Lençóis.

Despite the similarities between *E.delticus* and *E.bonariensis* measurements, morphological variations, and coloration (Eger 1977; Carter and Dolan 1978; Hunt et al. 2003; Gregorin et al. 2016), the differences described here for specimens assigned to these two species agree with the characters described by other authors (Marinkelle 1970; Bernardi et al. 2009) and further confirm these are distinct taxa. The specimens can be differentiated on the base of the following characters: posterior region of the skull more curved in *E.bonariensis*, with the interparietal bones not project posteriorly; incisive foramen diminutive in *E.bonariensis* and large in *E.delticus*; protocone of the second upper premolars wide and robust in *E.bonariensis*, in contrast to a thin and non-robust protocone in *E.delticus* (visible in lateral view of the skull); palate not extending beyond the level of the upper third molar in *E.bonariensis*, with the posterior part of this tooth extending beyond the maxillary bone; in *E.delticus* the palate surpasses the level of the upper third molar (Marinkelle 1970) and the posterior part of this tooth does not extend beyond the maxillary bone. In addition to these cranial features, the space between the basisphenoid pits is wide in *E.bonariensis* and narrow in *E.delticus*, although according to Bernardi et al. (2009) this character exhibits variability in the first species; however, the female of *E.bonariensis* reported here notably show the basisphenoid pits separated by a wider space with respect to *E.delticus*, such as is documented by this author.

The geographic distribution of *Eumopsbonariensis* in Brazil has been confirmed for the northeastern region in Bahia (but not in the Caatinga; Tavares et al. 2010; Ferreira et al. 2024), Paraná (Miretzki 2003), Rio Grande do Sul (Eger 1977; Pacheco and Freitas 2003; Bernardi et al. 2009; Medina et al. 2014; Gregorin et al. 2016), and São Paulo (Eger 1977; Medina et al. 2014; Ruelas and Soria 2021). Its global distribution also includes Argentina and Uruguay (Eger 2008). The female from the Caatinga extends the geographic range of this species 318 km NE from the nearest documented localities (Santana do Sobradinho, Carinhanha, 14°18'S, 43°45'W, Tavares et al. 2010).

Regarding *Eumopsdelticus*, the holotype was collected in Caldeirão, Marajó Island, Pará, Brazil (Eger 1977; Carter and Dolan 1978). Several authors previously considered this taxon as a subspecies or synonym of *E.bonariensis* (Sanborn 1932; Eger 1977) due to the morphological similarity of both taxa; however, it is recognized as a distinct species (Simmons and Cirranello 2024). Complementary specimens from Brazil, all of them examined and annotated by Eger (1977, 2008), Medina et al. (2014), and Gregorin et al. (2016), correspond to the following locations: Bahia (but not in the Caatinga) - São Marcelo and Ilha Madre de Deus; Pará-Boim, Tapajós River, and Marajó Island, Caldeirão; Amazonas-Faro, north bank of the Amazon River, Umaitá and Itacoatiara; and Minas Gerais-Uberlândia. The species is also known in southern Colombia and northern Peru. The female collected at Caatinga extends its geographic range 302 km NW from the nearest documented locality (Ilha Madre de Deus, 12°44'22.9"S, 38°36'47.0"W, Eger 2008).

Among the vouchers collected was a group of specimens with unequivocal characteristics that could only be identified to the genus *Molossus*. Defined species limits within this genus are unclear, with tenuous descriptions of many species and subspecies making the taxonomy of *Molossus* confusing and unstable (Loureiro et al. 2018b). The combination of characters of these specimens suggests a probable relation to *Molossusmolossus*. However, these specimens represent three clearly differentiated morphospecies requiring further evaluation to resolve species-level identification. Loureiro et al. (2018b) noted the possible presence of an undescribed species of *Molossus* in the Caatinga without further clarification of how it was differentiated from known species. In addition to those specimens noted above, a female with external and cranial features corresponding to the genus *Molossops* but not matching any known species suggests the possibility of an undescribed species differing from *M.temminckii* based on external and cranial features.

Previous information on the distribution of *Molossusaztecus* in Brazil indicated it occurred at three states within the Caatinga biome: Ceará, Piauí, and Pernambuco (Loureiro et al. 2018b); our records are the first for the Bahian Caatinga. A widespread species, *Molossusrufus* is known from seven of the 10 states within the Caatinga (Alagoas, Bahia, Ceará, Paraíba, Pernambuco, Minas Gerais, Piauí), based on historical records (Mares et al. 1981; Oliveira et al. 2003; Oliveira 2004; Astúa and Guerra 2008; Feijó and Langguth 2011) and recent information (Gurgel-Filho et al. 2015; Souza et al. 2016; Silva and Bernard 2017; Leal et al. 2022). Previous reports of this species in Bahia correspond to Carinhanha (Cerrado biome) and Atlantic Forest (Souza et al. 2016). Our records are the first verified occurrence of this species for the Bahian Caatinga.

Among the small bats of the genus *Molossus*, *M.molossus* was the most captured species in this study. Its distribution in the Caatinga is documented over an extensive area, including all federal units (Loureiro et al. 2018b). However, due to the considerable phenotypic variability of this species (Loureiro et al. 2018a, 2018b), identifications are often erroneously assigned to other species of similar size and morphology. Other species captured during this study have restricted distributions within Brazil or are poorly represented as vouchers in collections, e.g., *C.planirostris*, *E.glaucinus*, *N.laticaudatus*, *N.macrotis*, *M.aztecus*, and *M.rufus*. Our records for these species provide additional points contributing to a better understanding of these species distributions.

The sexual dimorphism found in the posterior lobe of the third upper molar morphology of *Molossopstemminckii* is especially interesting. This structure extends beyond the maxillary bone in the female, while in the male, it is less developed and does not extend beyond the maxillary bone; this has not been previously documented (Gamboa Alurralde and Díaz 2019). Prior to our records, the distribution of this species in the Caatinga included these states: Ceará (Mares et al. 1981; Oliveira et al. 2003; Oliveira 2004; Eger 2008; Gurgel-Filho et al. 2015; Feijó and da Rocha 2017), Minas Gerais (Pinto 2010), Paraíba (Leal et al. 2013; Beltrão et al. 2015), Pernambuco (Mares et al. 1981; Oliveira et al. 2003; Oliveira 2004), Piauí (Gregorin et al. 2008; Louzada et al. 2014), and Rio Grande do Norte (Silva and Bernard 2017; Vargas-Mena et al. 2018).

The absence of a sagittal crest in *Neoplatymopsmattogrossensis* was noted by Willig and Jones (1985), although Gurgel-Filho et al. (2015) indicated it was present in some specimens; however, they noted that this structure is difficult to see without magnification. Two individuals captured during this study had obvious sagittal crests, suggesting this is a variable trait within the species. This species has a broad geographical distribution in the Caatinga, with previous records reported from Alagoas (Leal et al. 2022), Ceará (Oliveira et al. 2003; Oliveira 2004; Eger 2008; Novaes et al. 2013; Gurgel-Filho et al. 2015; Feijó and da Rocha 2017), Paraíba (Leal et al. 2013), Pernambuco (Mares et al. 1981; Oliveira et al. 2003; Oliveira 2004; Astúa and Guerra 2008; Novaes et al. 2013; Carvalho-Neto et al. 2017), Piauí (Novaes et al. 2013), Rio Grande do Norte (Silva and Bernard 2017; Vargas-Mena et al. 2018), and Bahia (Oliveira et al. 2003; Oliveira 2004; Astúa and Guerra 2008; Eger 2008; Novaes et al. 2013).

Previously, *Nyctinomopslaticaudatus* was only known in the Caatinga from the Ceará (Feijó and Langguth 2011), Minas Gerais (Oliveira et al. 2003; Oliveira 2004), Paraíba (Feijó and Langguth 2011), Pernambuco (Mares et al. 1981; Oliveira et al. 2003; Oliveira 2004; Eger 2008; Astúa and Guerra 2008; Feijó and Langguth 2011; Carvalho-Neto et al. 2017), Piauí (Oliveira et al. 2003; Oliveira 2004), and one record of Bahia (Eger 2008). Our captures are the second record of the species in the Bahian Caatinga. *N.macrotis* has been reported from the Rio Grande do Norte (Vargas-Mena et al. 2018) and three locations in Bahia (Czaplewski and Cartelle 1998; Salles et al. 2014; Rocha et al. 2015). The single specimen we collected represents the fourth record of the species in the Bahian Caatinga, updating its distribution.

This study has increased the known number of species of Molossidae for the Caatinga biome to 21, with identifications verified by vouchers, including the confirmation of *E.bonariensis*, *E.delticus*, and *M.currentium* (in this study), *N.macrotis* (Czaplewski and Cartelle 1998; Salles et al. 2014; Rocha et al. 2015; Vargas-Mena et al. 2018), and *M.aztecus* (Loureiro et al. 2018b) not considered in the most recent species list (Silva et al. 2018) and *Cynomopsgreenhalli* recently reported from Paraíba and Pernambuco (Souza 2022).

The number could increase if a further analysis of the specimens listed here, classified only at the generic level, reveals new or additional taxa. The presence in our inventory of a relatively high number of species whose flight strategies make their detection difficult with conventional methods represents an important contribution, facilitated by the use of small lagoons as sampling sites, which many bats utilize for water consumption and, in some cases, for the capture of insects. Other studies have highlighted the importance of this methodological strategy for detecting Neotropical aerial insectivorous bats, which are usually considered cryptic (Ochoa-G 2000; Silva and Bernard 2017; Hintze et al. 2020).

As a complement to this survey, we included acoustic sampling, which also revealed a relatively high diversity of sonotypes, many of which were species of both Molossidae and Vespertilionidae. The taxonomic relationships of captures during this study to the acoustic sampling are being addressed in another publication. However, given the difficulty in capturing these elusive species, it is possible that some species that were acoustically recorded may have eluded capture during this study.

The data provided in the first stage of this research expand knowledge about the taxonomy and distribution of some members of the family Molossidae, including their presence in seasonal environments where dry forests conform the dominant vegetation. Future research is required to improve knowledge of this and other poorly studied Neotropical bat families, in order to provide information and insights for the design of conservation strategies (Cassano et al. 2017), in addition to a better representation of some taxonomic groups in scientific collections (Moras et al. 2018).

## ﻿Conclusions

This study provides a robust framework for future research on the ecology and conservation of bats in the Caatinga biome, thus contributing to the understanding and preserving biodiversity in this unique natural region. We further documented the diversity and importance of this biome as a habitat for species of molossids, with implications for biodiversity conservation. Additionally, our morphological assessments offer valuable insights into intraspecific variation and sexual dimorphism for several species, enhancing the understanding of their biology and evolution.

Our results underscore the need for continued research on such species’ morphological variations, ecological preferences, and conservation priorities. Finally, this study highlights the significance of small lakes or water bodies as sampling sites for documenting a high diversity of otherwise undersampled aerial insectivores.

## Supplementary Material

XML Treatment for Cynomops planirostris

XML Treatment for Eumops bonariensis

XML Treatment for Eumops delticus

XML Treatment for Eumops glaucinus

XML Treatment for Molossops temminckii

XML Treatment for Molossus aztecus

XML Treatment for Molossus currentium

XML Treatment for Molossus molossus

XML Treatment for Molossus rufus

XML Treatment for Neoplatymops mattogrossensis

XML Treatment for Nyctinomops laticaudatus

XML Treatment for Nyctinomops macrotis

## ﻿Additional information

### Author ORCIDs

Franger J. García https://orcid.org/0000-0001-6764-225X

José Ochoa-G https://orcid.org/0009-0000-4095-0463

José L. Poma-Urey https://orcid.org/0000-0002-2426-9819

Bruce W. Miller https://orcid.org/0000-0001-5719-1942

Fábio C. Falcão https://orcid.org/0000-0002-2748-7117

Martín Roberto del Valle Alvarez https://orcid.org/0000-0001-6908-8547

### Data availability

All of the data that support the findings of this study are available in the main text.
